# Ion Channel Blockers as Antimicrobial Agents, Efflux Inhibitors, and Enhancers of Macrophage Killing Activity against Drug Resistant *Mycobacterium tuberculosis*

**DOI:** 10.1371/journal.pone.0149326

**Published:** 2016-02-26

**Authors:** Diana Machado, David Pires, João Perdigão, Isabel Couto, Isabel Portugal, Marta Martins, Leonard Amaral, Elsa Anes, Miguel Viveiros

**Affiliations:** 1 Unidade de Microbiologia Médica, Instituto de Higiene e Medicina Tropical, IHMT, Universidade Nova de Lisboa, UNL, Lisboa, Portugal; 2 Global Health and Tropical Medicine, GHTM, Instituto de Higiene e Medicina Tropical, IHMT, Universidade Nova de Lisboa, UNL, Lisboa, Portugal; 3 iMed.ULisboa - Instituto de Investigação do Medicamento, Faculdade de Farmácia, Universidade de Lisboa, Lisboa, Portugal; 4 Instituto de Medicina Molecular, Faculdade de Medicina, Universidade de Lisboa, Lisboa, Portugal; 5 UCD Centre for Food Safety, School of Public Health, Physiotherapy and Sports Science, UCD Centre for Molecular Innovation and Drug Discovery, University College Dublin, Belfield, Dublin, Ireland; IPBS, FRANCE

## Abstract

Given the ability of *M*. *tuberculosis* to survive as an intracellular pathogen and its propensity to develop resistance to the existing antituberculosis drugs, its treatment requires new approaches. Here the antimycobacterial properties of verapamil, thioridazine, chlorpromazine, flupenthixol and haloperidol were investigated against a panel of drug resistant *M*. *tuberculosis* strains, both *in vitro* and on human-infected macrophages. These compounds are efflux inhibitors that share among them the characteristic of being ion channel blockers. *In vitro*, all compounds exhibited synergistic inhibitory activities when combined with isoniazid and rifampicin, and were able to inhibit active efflux, demonstrating their role as efflux inhibitors. Gene expression analysis showed that *M*. *tuberculosis* efflux genes were overexpressed in response to antibiotic exposure, *in vitro* and within macrophages, irrespective of their resistance pattern. These compounds displayed a rapid and high killing activity against *M*. *tuberculosis*, associated with a decrease in intracellular ATP levels demonstrating that the bactericidal action of the ion channel blockers against *M*. *tuberculosis* clinical strains is associated with their interference with energy metabolism. The compounds led to a decrease in the intracellular mycobacterial load by increasing phagosome acidification and activating lysosomal hydrolases. The results presented in this study enable us to propose the following mechanism of action for these compounds: a) in the bacteria, the compounds generate a cascade of events involving the inhibition of the respiratory chain complexes and energy production for efflux activity. Indirectly, this reduce the resistance level to antituberculosis drugs potentiating their activity; b) on the host cell, the treatment with the ion channel blockers increases phagosome acidification and induces the expression of phagosomal hydrolases, leading to bacterial growth restriction irrespective of their resistance pattern. This work highlights the potential value ion channel blockers as adjuvants of tuberculosis chemotherapy, in particular for the development of new therapeutic strategies, with strong potential for treatment shortening against drug susceptible and resistant forms of tuberculosis. Medicinal chemistry studies are now needed to improve the properties of these compounds, increasing their *M*. *tuberculosis* efflux-inhibition and killing-enhancement activity and reduce their toxicity for humans, therefore optimizing their potential for clinical usage.

## Introduction

*Mycobacterium tuberculosis* is a pathogen difficult to control mostly due to its impenetrable cell wall coupled with a long generation time, a plastic metabolism and a remarkable ability to establish persistent infections. Therefore, the therapeutic regimen for tuberculosis requires a prolonged antibiotic treatment in order to attain a favourable clinical outcome. Paradoxically, the longtime needed for the therapeutic regimen is also a critical obstacle for elimination of the pathogen due to frequent patient non-compliance to the treatment. Consequently, there will be an increased probability of developing first-line drug resistant forms of tuberculosis, which requires a less effective, more toxic and even more prolonged second-line therapeutic regimen [1]. The few new drugs in the pipeline urges the need for new and better time-efficient approaches for the treatment of tuberculosis, in particular for the drug resistant forms [1; 2].

Nowadays is widely accepted that efflux mechanisms contribute to the overall antibiotic resistance in *M*. *tuberculosis*. Efflux pump activity contributes to drug resistance by allowing the bacteria to survive for a longer period of time in the presence of subinhibitory concentrations of antibiotics, until mutations for resistance emerge and are established in the bacterial population [3; 4]. This prolonged exposure under antibiotic pressure increases the probability of selection of spontaneous mutants with high-level resistance [3–6]. An approach to prevent these events from occurring is the inhibition of efflux pumps. Several compounds have been appointed as putative bacterial efflux inhibitors. Among these are the calcium channel blocker of eukaryotic cells verapamil; the phenothiazines such as thioridazine, a class of antipsychotics drugs; natural-derived compounds such as reserpine, among others. These compounds have been shown to have an antimicrobial effect against susceptible *M*. *tuberculosis in vitro* and in the macrophage model [7–10]. Nevertheless, the mechanism by which these compounds are active against *M*. *tuberculosis* is not fully understood. A common hypothesis has been postulated for the observed direct and indirect activity of these compounds against *M*. *tuberculosis*: they enhance the inhibitory activities of antituberculosis drugs *in vitro* and enhance the killing activity of the macrophage against intracellular *M*. *tuberculosis*, being potential adjuvants in tuberculosis chemotherapy [2; 11; 12]. In this study, we aimed to study the mechanism of action of ion channel blockers both *in vitro* and on human macrophages against multi and extensively drug resistant *M*. *tuberculosis* clinical strains. This was done by testing (i) the synergistic effect of the compounds in combination with first- and second-line antituberculosis drugs using the MGIT system to attain clinical correlation; ii) their ability to inhibit ethidium bromide efflux activity, as direct evidence of their efflux inhibitory activity; iii) their influence on energy metabolism by the evaluation of bacterial intracellular ATP levels after exposure to the compounds; iv) their antimycobacterial and synergistic effect in combination with isoniazid and rifampicin on the macrophage model, and v) their effect in vacuolar acidification and activation of hydrolases. The unravelling of the mechanism of action of the ion channel blockers verapamil, thioridazine, chlorpromazine, flupentixol and haloperidol, that allows them to act as antimicrobial agents, efflux inhibitors and enhancers of macrophage killing activity against *M*. *tuberculosis*, provides new insights of their potential as future adjuvants in tuberculosis chemotherapy, as well as identify some of the mediators for their antimycobacterial and efflux inhibitory activity.

## Materials and Methods

### Mycobacterial strains, antibiotics and chemicals

The *M*. *tuberculosis* strains used in this study were obtained from the culture collection maintained at the Mycobacteriology Laboratory (Instituto de Higiene e Medicina Tropical, Universidade Nova de Lisboa) and comprised the *M*. *tuberculosis* H37Rv ATCC27294^T^, *M*. *tuberculosis* H37Rv_ΔkatG_ [3] and seven drug-resistant clinical strains (Table 1). *Mycobacterium bovis* BCG Pasteur ATCC35734 expressing GFP (BCG-GFP) was kindly provided by Prof. M. Niederweis (University of Alabama at Birmingham, USA). Isoniazid, rifampicin, ofloxacin, amikacin, capreomycin, verapamil, flupenthixol, thioridazine, chlorpromazine, haloperidol, and the efflux substrate ethidium bromide were obtained from Sigma-Aldrich (St. Louis, MO, USA).

**Table 1:** Molecular characterization,resistance pattern and MIC values of the antibiotics and inhibitors for the nine *M. tuberculosis*strains.

				Antibiotics (μg/ml)				Inhibitors (μg/ml)				
Strain	Resistance pattern	Gene mutations	INH	RIF	AMK	CAP	OFX	VP	TZ	CPZ	FPX	HAL
**H37Rv**	Susceptible	None	0.1	1	1	2.5	1	256	15	30	30	64
**H37Rv**_**ΔkatG**_	INH	*katG* deletion	128	1	1	2.5	1	128	15	30	30	32
**82/09**	INH; RIF–(MDR)	*inhA* C-15T/S94A; *rpoB* S531L	3	320	1	2.5	1	256	15	30	30	64
**149/09**	INH; RIF; AMK; CAP; OFX– (XDR)	*inhA* C-15T/I194T; *rpoB* 531L; *gyrA* D94A; *rrs* A1401G	3	320	640	25	10	256	15	30	30	64
**286/09**	INH; RIF; AMK; CAP; OFX–(XDR)	*inhA* C-15T/S94A; *rpoB* S531L; *gyrA* S91P; *eis* G-10A; *tlyA* ins GT at pos 755/756	20	80	4	25	10	128	15	30	30	32
**69/11**	INH; RIF; AMK; CAP–(MDR)	*katG* S315T; *rpoB* S531L; *rrs* A1401G/wt*	20	320	40	25	1	256	15	30	30	32
**29/12**	INH; RIF; AMK; CAP; OFX–(XDR)	*inhA* C-15T/I194T; *rpoB* S531L; *gyrA* D94A; *rrs* A1401G	3	320	640	>25	10	256	15	30	30	64
**269/03**	INH	*katG* S315T	10	1	1	2.5	1	256	15	30	30	64
**294/09**	INH	*inhA* C-15T	0.4	1	1	2.5	1	256	15	30	30	64

Δ, deletion; MDR, multidrug resistant; XDR, extensively drug resistant; INH, isoniazid; RIF, rifampicin; AMK, amikacin; CAP, capreomycin; OFX, ofloxacin; VP, verapamil; TZ, thioridazine; CPZ, chlorpromazine; FPX, flupenthixol; HAL, haloperidol.

*mixed pattern: simultaneous presence of drug susceptible and drug resistant genotype. The lowest concentration tested corresponds to the critical concentration for each antibiotic (see Material and Methods section for details).

### Susceptibility testing

#### (i) First- and second-line drug susceptibility testing

The BACTEC MGIT 960 system (MGIT960) was used for first- and second-line drug susceptibility testing according to the manufacturer’s instructions. MGIT tubes were inoculated with 0.8 ml SIRE supplement (Becton Dickinson), 0.1 ml of antibiotic at the critical concentrations and 0.5 ml of the suspension of the strain. For preparation of the drug-free proportional control strain suspension was diluted 1:100 (1:10 for PZA) and 0.5 ml inoculated in the tube. Antibiotics were tested at the critical concentration as follows: 0.1 μg/ml for isoniazid, 1 μg/ml for rifampicin, 1 μg/ml for streptomycin, 5 μg/ml for ethambutol, 100 μg/ml for pyrazinamide, 1 μg/ml for amikacin, 2.5 μg/ml for capreomycin, and 1 μg/ml for ofloxacin [13]. The results were interpreted according to manufacturer’s instructions. Briefly, at the time of positivity of the proportional control (Growth units [GU] = 400), the comparison between this tube and the tubes containing the drugs(s) was performed. If the GU of the tubes containing the drug were >100, they were considered to be resistant to that concentration. If the GU of the tube containing the drug was <100 they were considered susceptible [14]. Growth of the cultures was monitored with the Epicenter V5.80A software equipped with the TB eXIST module.

#### (ii) MIC determination of antibiotics and inhibitors

MIC determination was done within MGIT960 and the growth monitored with the Epicenter V5.80A software. Verapamil and haloperidol were tested at concentrations ranging from 16 to 512 μg/ml; thioridazine, chlorpromazine and flupenthixol were tested at concentrations from 3.75 to 60 μg/ml. Isoniazid was tested concentrations ranging from 0.1 to 120 μg/ml, rifampicin and amikacin were tested from 1 to 640 μg/ml and ofloxacin was tested from 1 to 16 μg/ml. At the time of testing, two-fold serial dilutions were prepared to achieve the desired concentrations. When growth was observed in the drug containing tube (GU≥100) before the proportional growth control (1% inoculum) was positive, this indicates that more than 1% of the population was growing in the presence of the drug and as per proportion testing, the strain is considered resistant at the corresponding drug concentration [13]. Thus, the MIC was considered as the lowest concentration with GU <100 when the drug-free control tube reached the positivity threshold of 400 GU.

### Quantitative drug susceptibility testing of antibiotics in the presence and absence of inhibitors

Quantitative drug susceptibility testing of rifampicin, isoniazid, ofloxacin and amikacin was conducted using the MGIT960 and the Epicenter V5.80A/TB eXIST. Isoniazid was tested at 0.1, 1, 3 and 10 μg/ml; rifampicin and amikacin at 1, 4 and 20 μg/ml; and ofloxacin at 1, 2 and 10 μg/ml. The interpretation of the results was performed as per Springer et al [14]. The quantification of the resistance levels was done according to Cambau et al [13] as follows: isoniazid low-level resistance when resistant (R) at 0.1 and susceptible (S) at 1 μg/ml; isoniazid high-level resistance when R ≥ 1; rifampicin and amikacin low-level resistance when R at 4 and S at 20 μg/ml; rifampicin and amikacin high-level resistance when R ≥ 20 μg/ml; ofloxacin low-level resistance when R at 1 and S at 2 μg/ml; ofloxacin high-level resistance when R ≥ 2 μg/ml. The preparation of the drug containing tubes and controls was done as described above. For the susceptibility testing for the antibiotics in the presence of the inhibitors, the tubes containing 0.1 ml of the antibiotics at the desired concentrations were inoculated with 0.1 ml of the inhibitor to a final concentration of half MIC. This concentration was selected since it has no effect on the growth of the strains following the protocol described above. The resistance levels for each combination (antibiotic + efflux inhibitor) were quantified as describe above.

### Detection of mutations associated with resistance

Genomic DNA was extracted using the QIAamp DNA mini kit (QIAGEN) according to the manufacturer’s instructions. The most common mutations in *katG* gene and *inhA* regulatory region were investigated using the Genotype MTBDR*plus* (Hain Lifescience GmbH, Nehren, Germany). For the detection of the most frequent mutations in *gyrA* and *rrs* genes, the Genotype MTBDR*sl* (Hain Lifescience) was used according to the manufacturer’s instructions. Genomic analysis of the complete *inhA* and *tlyA* gene,and *eis* promoter region was performed by PCR amplification and DNA sequencing using the primers and conditions described previously [15–17].

### Efflux pump gene expression

#### (i) Sample preparation

For the analysis of efflux pump gene expression upon exposure to isoniazid and rifampicin, each strain was inoculated into MGIT tubes containing 10% OADC plus isoniazid or rifampicin at the respective MIC (see Table 1 for MIC values). The MIC for *M*. *tuberculosis* is the MIC 99% where only less than one percent of the bacterial population is allowed to survive the exposure to that concentration after a defined period of time (maximum of 12 days within the MGIT 960 liquid media). The strains were then incubated at 37°C within the BACTEC system until growth was achieved as previously described [3]. At the concentrations used, 12 to 30 days of extended incubation after positivity of the proportional growth control tube, growth on the tubes containing the antibiotic at the MIC was obtained for both antibiotics and for all strains. At this point, samples of each culture were taken for RNA extraction and RT-qPCR analysis. For the evaluation of *M*. *tuberculosis* efflux pump gene expression from infected macrophages, human monocyte-derived macrophages were infected with *M*. *tuberculosis* strain 82/09 at a MOI 1 and were allowed to uptake the bacteria for three hours. Cells were then washed three times with phosphate buffered solution (PBS) to remove extracellular bacteria and treated with isoniazid at 0.1 μg/ml (critical concentration). At day three post-infection, cells were lysed with 0.05% Igepal (Sigma-Aldrich) and RNA extracted from macrophage-grown bacilli as described below.

#### (ii) RNA extraction

Total RNA was isolated from cultures and *M*. *tuberculosis*-infected macrophages using a GTC/Trizol based method. Briefly, five volumes of a 5M GTC solution were added to each sample and incubated at room temperature for five minutes. Mycobacteria were harvested by centrifugation and the pellet resuspended in 1 ml of TRI reagent (Sigma). The mixture was transferred to lysis tubes containing glass beads (QIAGEN, GmbH, Hilden, Germany) and sonicated at 35 kHz (Gen-Probe, California, USA) during three x five minutes with one minute of cooling on ice between intervals. After processing, 300 μl of chloroform: isoamyl alcohol (24:1) was added to the mixture. The top aqueous layer was separated by centrifugation, transferred to a clean tube containing an equal volume of isopropanol, and incubated overnight at -20°C. Precipitated nucleic acids were recovered by centrifugation, washed twice with 70% ice-cold ethanol, incubated with an RNase-free DNase I (QIAGEN) during 30 minutes, followed by phenol extraction. Samples were incubated overnight at -20°C, washed twice with 70% ice-cold ethanol, dried, and dissolved in RNase-free water. Quantity and quality of the purified RNA was measured using a NanoDrop 1000 spectrophotometer (Thermo Scientific, Waltham, USA). Gene expression levels were analysed by RT-qPCR in the strains exposed to the MIC of isoniazid or rifampicin. The primers employed are described in the supporting information (S1 Table). The RT-qPCR procedure was performed in a Rotor-Gene 3000 thermocycler (Corbett Research, Sidney, Australia) and followed the protocol recommended for use with the QuantiTect SYBR Green RT-PCR Kit (QIAGEN) as described previously [3]. The relative expression of the genes was determined by comparison of the relative quantity of the respective mRNA in the presence of the antibiotic to the non-exposed condition using the comparative quantification 2^-ΔΔCt^ method [18]. A level of relative expression equal to 1 indicates that the expression level was identical to the non-exposed strain. Genes showing expression levels above one, when compared with the non-exposed strain, were considered to be overexpressed. Relative expression levels above two were considered significantly overexpressed. Each strain was assayed in triplicate using total RNA obtained from three independent cultures. Data was normalized to the *M*. *tuberculosis* 16S rDNA reference gene and presented as the mean fold change (±SD) compared with the control. The same method and procedures were used to quantify efflux pump gene expression of *M*. *tuberculosis* in response to phagocytosis by cultured human primary macrophages.

### Assessment of efflux activity

The detection of ethidium bromide efflux was performed by a semi-automated fluorometric method as described before [19] with a modified protocol for *M*. *tuberculosis* [3]. Briefly, the strains were grown in 10 ml of MB7H9 medium containing 10% OADC enrichment and 0.05% Tween 80, at 37°C, until they reached an OD_600_ of 0.8. After this, bacterial cells were collected by centrifugation and the pellet washed twice in PBS. In order to determine the lowest concentration of ethidium bromide that causes accumulation (equilibrium concentration), the bacterial cells (final OD_600_ 0.4) were incubated with different concentrations of ethidium bromide plus 0.4% glucose. Fluorescence was then measured, at 37°C, in a Rotor-Gene 3000 thermocycler (excitation, 530 nm; emission, 585 nm). To evaluate the effect of the inhibitors on the accumulation of ethidium bromide, the assays were performed as described above with the exception that each efflux inhibitor was added to the solution at half MIC and ethidium bromide was used at the equilibrium concentration (determined for each strain). The effect of the efflux inhibitors was evaluated by the determination of the relative final fluorescence (RFF) as described previously [19; 20]. Each assay was performed in triplicate and the results presented correspond to the average of three independent assays (±SD). For the efflux assays, the bacterial cells were incubated with verapamil at half MIC and ethidium bromide at the equilibrium concentration for one hour at room temperature. Next, bacterial cells were collected by centrifugation, washed and resuspended in fresh buffer with and without 0.4% glucose in the presence and absence of half MIC of verapamil, and the fluorescence measured as described above.

### Time-kill kinetics

Time-kill assays were performed according to Warman et al [21]. Mid-log phase cultures were diluted to 1×10^5^ CFU/ml and challenged with the compounds and antibiotics at 5X their MIC. A drug-free control was included in the assay to monitor the normal growth of the strains. Cultures were sampled for CFU determination after one, two, and three days of incubation at 37°C.

Colony forming units were determined with the MGIT960 and the Epicenter software. Briefly, to generate calibration curves, the number of viable bacterial cells was first determined by the standard plate counting method. Tenfold serial dilutions were made in MB7H9 and 100 μl from each dilution plated onto MB7H11 plates (DIFCO) supplemented with 10% OADC. Plates were sealed and the CFU’s determined after incubation at 37°C during 21 days. Simultaneously, MGIT tubes supplemented with 10% OADC were inoculated in triplicate with 100 μl of the same serial dilutions, incubated in the MGIT960 system and the time to detection (TTD) recorded. Calibration growth curves for each dilution were generated with the Epicenter software and the TTD for each dilution plotted against log_10_ CFU. To obtain the bacterial cell concentration for each drug-exposed culture, CFU’s were predicted by the theoretical log_10_ CFU based TTD multiplied by the corresponding dilution factor. The limit of detection of the assay was 1 CFU/ml. The killing effect of the compounds on *in vitro* growth of *M*. *tuberculosis* was defined as the lack of growth in MGIT tubes after 100 days of incubation [22].

### Quantification of intracellular ATP

Intracellular ATP was quantified by using the ATP Determination Kit (Invitrogen, Life Technologies, Paisley, UK) according to the manufacturer’s instructions. Briefly, strains were exposed to the compounds and at various time points, aliquots of 1 ml of bacteria were collected, bacterial cells were harvested by centrifugation, the supernatant discarded and the pellet resuspended in 1 ml of MB7H9. Samples were heat inactivated and immediately deep-frozen. Bacterial lysates were transferred into white flat bottom 96-well plates and the ATP content measured using a Tecan’s Infinite M200 plate reader (Tecan Trading AG, Switzerland) and expressed as relative luminescence units. Isoniazid and rifampicin were used as controls. Cultures were sampled for CFU determination as described above.

### Antimycobacterial activity on infected macrophages

#### (i) Mycobacterial cultures

Mycobacterial strains were cultured in MB7H9 supplemented with 10% OADC and 0.05% tyloxapol (Sigma-Aldrich) at 37°C. For BCG-GFP, 50 μg/ml of hygromycin (Sigma-Aldrich) were added to the media. Bacterial cultures on exponential growth phase were collected by centrifugation, washed in PBS and resuspended in cell culture medium without antibiotics.

#### (ii) Isolation and culture of human monocyte-derived macrophages

Human monocyte-derived macrophages were obtained from buffy coat preparations kindly donated by Instituto Português do Sangue, Portugal. The buffy coat was diluted in PBS containing 0.5% bovine serum albumin and 2 mM of EDTA and overlaid on Ficoll-Paque Plus (GE Healthcare, Freiburg, Germany) at a ratio of 2:1 followed by 800 x *g* centrifugation for 20 minutes at room temperature. The interface was recovered and washed two times with PBS. Selection of CD14 monocytes was performed using MACS LS cell separation system (MiltenyiBiotec, Cologne, Germany) according to the manufacturer’s instructions. CD14 monocytes were differentiated into macrophages during seven days in macrophage medium containing RPMI-1640 medium with 10% fetal bovine serum, 1% GlutaMAX, 1 mM sodium pyruvate, 10 mM HEPES at pH 7.4, 100 IU/ml penicillin and 100 μg/ml streptomycin (Gibco, Life Technologies), 20 ng/ml of macrophage colony-stimulating factor (Immunotools, Friesoythe, Germany) and incubated at 37°C with 5% CO_2_. Fresh medium was added after four days of isolation.

#### (iii) Determination of macrophage viability after treatment with compounds

Human monocyte-derived macrophages were seeded at 3x10^5^ cells per well in 24-well plates and treated with the compounds. After three days of treatment, cell viability was determined using AlamarBlue (Molecular Probes, Life Technologies) following the manufacturer’s indications. For the subsequent intracellular assays, the compounds were used at concentrations that were shown to be nontoxic for the macrophages: verapamil, 10 μg/ml; thioridazine, 2.5 μg/ml; chlorpromazine, flupenthixol and haloperidol, 1.25 μg/ml (S1 Fig). Complementing our previous studies, where 0.1 μg/ml of thioridazine was shown to be sufficient to enhance the killing activity of *M*. *tuberculosis* human-infected macrophages, but well below the clinical dose [9], we have now selected a clinically more relevant concentration, 2.5 μg/ml. This concentration was shown to be non-cytotoxic for the macrophages and yet is well within the maximum therapeutic ranges, considering that the usual maximum dose of thioridazine which is in excess of 600 mg/day. Isoniazid and rifampicin were used at the critical concentrations of 0.1 μg/ml and 1 μg/ml, respectively.

#### (iv) Quantification of intracellular bacterial survival

Human monocyte-derived macrophages were infected with *M*. *tuberculosis* strains at an MOI 1 and were allowed to uptake the bacteria for three hours. Cells were then washed three times with PBS and treated with the compounds. At day three post-infection, cells were lysed with 0.05% Igepal. Serial dilutions of the lysate were plated on MB7H11 medium supplemented with 10% OADC. Colony forming units were counted upon three weeks of incubation at 37°C.

#### (v) Quantification of compound-induced macrophage acidification

Human monocyte-derived macrophages were infected with BCG-GFP at a MOI 10, which was previously shown to provide optimum phagocytosis in these experimental conditions [23], and were allowed to uptake the bacteria for three hours. After, the cells were washed and treated with the compounds during 24 hours at 37°C with 5% CO_2_. At 24 hours post-infection, cells were incubated with LysoSensor Yellow/Blue DND-160 (Molecular Probes) at 1 μM for 10 minutes, washed and observed under a LSM 510 META laser scanning confocal microscope (ZEISS, Germany). LysoSensor Yellow/Blue DND-160 emits yellow/dark green fluorescence in neutral environments, but changes to green/blue in more acid environments. Quantification of acidified cells by flow cytometry was performed by detaching the cells with 0.05% Trypsin-EDTA (Sigma-Aldrich). After this, cells were washed with PBS and incubated with 100 nM LysoTracker Red DND-99 (Molecular Probes) for 30 minutes at 37°C and 5% CO_2_. Cells were analysed on an easyCyte 5HT flow cytometer (Millipore Corporation, Billerica, MA, USA), with green and yellow fluorescence emission off the blue laser filter (GRN-B and YEL-B), and with a LSM 510 META laser scanning confocal microscope. For confocal microscopy were used laser lines at 488 nm for GFP, 543 nm for LysoTracker Red and 364 nm for LysoSensor Yellow/Blue. Emission was detected with a band-pass filter of 505–550 nm for GFP, 565–615 nm for LysoTracker Red and 450–520 nm for LysoSensor Yellow/Blue. Image acquisition was carried out using an X63 (1.4 numerical aperture) plan apochromat oil-immersion objective. Images were acquired using a LSM 510 imaging software and analyzed with Image J software (National Institutes of Health, Bethesda, Maryland, USA).

#### (vi) Measurement of cathepsin B activity

Human monocyte-derived macrophages were infected with *M*. *tuberculosis* H37Rv at an MOI 1 for three hours. The cells were then incubated with the compounds during 24 hours at 37°C with 5% CO_2_. Cathepsin B activity was measured using a fluorometric cathepsin B activity assay kit (BioVision, California, USA) in accordance with manufacturer's instructions.

### Statistical analyses

Statistical analysis was performed with GraphPad Prism V5.01 software (La Jolla, USA). For comparisons between groups was used Student’s t-test. A *P* value < 0.05 was considered statistically significant (two-tailed tested).

## Results

### Ion channel blockers reduce the resistance level of first- and second-line antituberculosis drugs

The molecular characterization, resistance pattern, and MIC values of the antibiotics and inhibitors for the nine *M*. *tuberculosis* strains is presented in Table 1. The effect of the inhibitors on the levels of resistance to isoniazid, rifampicin, amikacin, and ofloxacin is presented in Table 2. The resistance levels to isoniazid were reduced from high-level to low-level in 4/8 of the resistant strains, with verapamil, flupenthixol, chlorpromazine and haloperidol, and 2/8 with thioridazine. None of the strains showed reversal of resistance since the resistance levels did not drop below the isoniazid critical concentration (0.1 μg/ml) (Table 2). The four strains for which the resistance level was reduced in the presence of the inhibitors harboured double mutations in *inhA* gene, two with C-15T/S94A and two with C-15T/I194A substitutions. The four remaining strains, for which residual or no change of the resistance level was noticed in the presence of the inhibitors, harboured single mutations: the C-15T mutation in *inhA* promoter in one strain, two strains with the S315T mutation in *katG*, and one with a complete deletion of *katG*. Regarding the strains with the *katG* S315T mutation, only a slight enhancement of the antimicrobial effect of isoniazid was observed for both strains in the presence of thioridazine, chlorpromazine and flupenthixol (Table 2). However, this potentiation was not enough to reach low-level isoniazid resistance. Regarding strain 294/09 with the C-15T mutation in the promoter region of *inhA* gene, its low-level resistance is due to the overexpression of *inhA* and the inhibitors tested could not overcome the effect of this overexpression on isoniazid resistance. Concerning rifampicin, five strains presented high-level resistance to this antibiotic due to the presence of the mutation S531L in *rpoB*. The resistance level was remarkably reduced from high- to low-level in the presence of verapamil (5/5), chlorpromazine (4/5), and thioridazine, flupenthixol and haloperidol (1/5) (Table 2). Four strains presented resistance to amikacin, three of them due to a mutation in the 1400 region of *rrs* and one strain in the *eis* promoter region. The high-level resistance conferred by mutations in *rrs* could not be reduced in the presence of the inhibitors in 2/3 resistant strains. Nevertheless, resistance to amikacin was reversed in strain 69/11 by each of the five compounds. In this case, the *rrs* hybridization pattern given by the Genotype MTBDR*sl* assay indicated heteroresistance. Amikacin resistance due to the mutation in *eis* promoter region could not be reduced. Again, resistance due to the overexpression of the target gene for resistance cannot be altered. For ofloxacin, low-level resistance was detected in three strains and correlated with mutations in *gyrA* gene. With this panel of strains, ofloxacin resistance could not be reduced by any of the inhibitors tested. These results indicate that the overall level of resistance of these strains cannot be due only to the presence of mutations in the drug targets but also has a significant contribution from a physiological response from the bacteria once in presence of the antibiotics, most probably due to the activity of efflux pumps.

**Table 2:** Synergistic activity between the ion channel blockers and isoniazid,rifampicin,amikacin or ofloxacin,against the nine *M. tuberculosis*strains.

		Strains																	
Drug combinations		H37Rv—Suscep		H37Rv_ΔkatG_—INH^R^		82/09—MDR		149/09—XDR		286/09—XDR		69/11—MDR		29/12—XDR		269/03—INH^R^		294/09—INH^R^	
Antibiotics	ICB	MIC (μg/ml)	MF	MIC (μg/ml)	MF	MIC (μg/ml)	MF	MIC (μg/ml)	MF	MIC (μg/ml)	MF	MIC (μg/ml)	MF	MIC (μg/ml)	MF	MIC (μg/ml)	MF	MIC (μg/ml)	MF
**INH**	**No ICB**	0.1	-	128	-	3	-	3	-	20	-	20	-	3	-	10	-	0.4	-
	**+ VP**	0.1	1	128	1	**1**	**3**	**1**	**3**	**1**	**20**	**10**	**2**	**1**	**3**	10	1	0.4	1
	**+ TZ**	0.1	1	128	1	**1**	**3**	**1**	**3**	20	1	**3**	**7**	3	1	**3**	**3**	0.4	1
	**+ CPZ**	0.1	1	128	1	**1**	**3**	**1**	**3**	**1**	**20**	**3**	**7**	**1**	**3**	**3**	**3**	0.4	1
	**+ FPX**	0.1	1	128	1	**1**	**3**	**1**	**3**	**1**	**20**	**3**	**7**	**1**	**3**	**3**	**3**	0.4	1
	**+ HAL**	0.1	1	128	1	**1**	**3**	**1**	**3**	**1**	**20**	**10**	**2**	**1**	**3**	10	1	0.4	1
**RIF**	**No ICB**	1	-	-	-	320	-	320	-	80	-	320	-	320	-	-	-	-	-
	**+ VP**	1	1	**-**	**-**	**20**	**16**	**20**	**16**	**20**	**4**	**20**	**16**	**20**	**16**	**-**	**-**	**-**	**-**
	**+ TZ**	1	1	**-**	**-**	320	1	320	1	**20**	**4**	320	1	320	1	**-**	**-**	**-**	**-**
	**+ CPZ**	1	1	**-**	**-**	320	1	**20**	**16**	**4**	**20**	**20**	**16**	**20**	**16**	**-**	**-**	**-**	**-**
	**+ FPX**	1	1	**-**	**-**	320	1	320	1	**4**	**20**	320	1	320	1	**-**	**-**	**-**	**-**
	**+ HAL**	1	1	**-**	**-**	320	1	320	1	**20**	**4**	320	1	320	1	**-**	**-**	**-**	**-**
**AMK**	**No ICB**	1	-	-	-	-	-	640	-	4	-	40	-	640	-	-	-	-	-
	**+ VP**	1	1	**-**	**-**	**-**	**-**	640	1	4	1	**1**	**40**	640	1	**-**	**-**	**-**	**-**
	**+ TZ**	1	1	**-**	**-**	**-**	**-**	640	1	4	1	**1**	**40**	640	1	**-**	**-**	**-**	**-**
	**+ CPZ**	1	1	**-**	**-**	**-**	**-**	**40**	**16**	4	1	**1**	**40**	**40**	**16**	**-**	**-**	**-**	**-**
	**+ FPX**	1	1	**-**	**-**	**-**	**-**	640	1	4	1	**1**	**40**	640	1	**-**	**-**	**-**	**-**
	**+ HAL**	1	1	**-**	**-**	**-**	**-**	640	1	4	1	**1**	**40**	640	1	**-**	**-**	**-**	**-**
**OFX**	**No ICB**	1	-	**-**	**-**	**-**	**-**	10		10		**-**	**-**	10		**-**	**-**	**-**	**-**
	**+ VP**	1	1	**-**	**-**	**-**	**-**	10	1	10	1	**-**	**-**	10	1	**-**	**-**	**-**	**-**
	**+ TZ**	1	1	**-**	**-**	**-**	**-**	10	1	10	1	**-**	**-**	10	1	**-**	**-**	**-**	**-**
	**+ CPZ**	1	1	**-**	**-**	**-**	**-**	10	1	10	1	**-**	**-**	10	1	**-**	**-**	**-**	**-**
	**+ FPX**	1	1	**-**	**-**	**-**	**-**	10	1	10	1	**-**	**-**	10	1	**-**	**-**	**-**	**-**
	**+ HAL**	1	1	**-**	**-**	**-**	**-**	10	1	10	1	**-**	**-**	10	1	**-**	**-**	**-**	**-**

Suscep, susceptible; MF, modulation factor; ICB, ion channel blocker; Δ, deletion; INH, isoniazid; RIF, rifampicin; AMK, amikacin, OFX, ofloxacin; VP, verapamil; TZ, thioridazine; CPZ, chlorpromazine; FPX, flupenthixol; HAL, haloperidol. The lowest concentration tested corresponds to the critical concentration for each antibiotic (see Material and Methods section for details). Synergistic interactions are in bold.

### Overexpression of efflux pumps in response to the antibiotic pressure leads to increased tolerance towards antibiotics

To assess the contribution of the *M*. *tuberculosis* membrane transporters for the increased levels of resistance, the two most effective antituberculosis drugs, isoniazid and rifampicin, where chosen to be tested as inducers of efflux pump overexpression and activity. The strains were exposed to isoniazid or rifampicin at their MICs (for *M*. *tuberculosis* this is the concentration that allows less than 1% of the population to grow in the presence of a drug *as per* the proportion method) and the expression levels of genes that code for 10 efflux pumps and the global regulator *whiB7* were evaluated by RT-qPCR (Tables 3 and 4). As can be observed, the majority of the genes are overexpressed in response to the exposure to isoniazid or rifampicin, nevertheless, without a specific pattern of expression. Regarding the exposure to rifampicin, increases in the expression of the majority of the genes tested are evident, albeit at lower absolute levels than those obtained for the isoniazid exposed strains. The systematic increase in efflux pump gene expression after antibiotic exposure may be explained by two possible mechanisms: a) increase in gene expression due to a mutation in the promoter region of the efflux pump gene, leading to constitutive gene expression; and b) induction promoted by the compounds *via* global regulators. Increase in gene expression due to a mutation in a promoter region of the efflux pump gene is less likely to occur since we found no overexpression of any of these genes without antibiotic treatment in the strains studied (data not shown). Nevertheless, to clarify which mechanism is promoting this overexpression, we searched for mutations on the transporter genes studied. Despite the detection of some missense or silent mutations in the coding regions of these genes, no other mutations were found in the putative promoter or regulatory regions of these strains (S2 Table). This indicates that the overexpression detected in these strains *in vitro* is most probably a global response mediated by global regulators. Although the expression levels observed for *whiB7* upon antibiotic exposure were moderate, they do not exclude the hypothesis of a global cellular response to the antibiotic stress, involving *whiB7* or another regulator.

**Table 3:** Average quantification of the relative expression level,by RT-qPCR,of the genes that code for efflux pumps in *M. tuberculosis*exposed to isoniazid.

	Relative expression level ± SD						
Gene	82/09—MDR	149/09—XDR	286/09—XDR	29/12—XDR	69/11—MDR	294/09—INH^R^	269/03—INH^R^
***mmr***	**6.29 ± 3.23**	**2.22 ±0.11**	0.78 ± 0.22	**1.63 ± 0.16**	0.76 ± 0.07	0.64 ± 0.03	0.74 ± 0.04
***mmpL7***	0.49 ± 0.38	**2.49 ± 0.49**	**2.23 ± 0.33**	**4.03** ± 2.87	0.76 ± 0.00	0.83 ± 0.24	0.72 ± 0.21
***Rv1258c***	0.79 ± 0.40	**1.34 ± 0.26**	0.48 ± 0.40	**2.22 ± 0.11**	0.79 ± 0.30	0.85 ± 0.12	0.64 ± 0.03
***p55***	**26.06 ± 2.55**	**1.65 ± 0.92**	0.67 ± 0.13	**4.04** ± 0.78	0.86 ± 0.41	**1.38 ± 0.20**	0.69 ± 0.17
***whiB7***	**3.38 ± 1.29**	0.19 ± 0.01	0.71 ± 0.07	0.87 ± 0.00	0.61 ± 0.15	0.67 ± 0.13	**2.55** ± 1.32
***Rv1217c***	**5.94 ± 1.44**	0.08 ± 0.01	0.60 ± 0.30	**5.12** ± 1.96	0.69 ± 0.17	**1.33 ± 0.26**	0.62 ± 0.06
***Rv1218c***	**1.36 ± 0.91**	0.04 ± 0.00	0.50 ± 0.00	**2.17 ± 0.42**	0.44 ± 0.08	**1.28 ± 0.06**	0.72 ± 0.13
***efpA***	**10.11 ± 3.82**	**1.38 ± 0.21**	0.80 ± 0.18	**1.76 ± 0.34**	**1.39** ± 0.33	0.81 ± 0.00	**1.52** ± 0.15
***Rv2459***	0.44 ± 0.15	**1.62 ± 0.00**	0.44 ± 0.26	**1.86 ± 0.63**	0.68 ± 0.19	0.73 ± 0.04	0.94 ± 0.09
***pstB***	**1.54 ±0.66**	**1.12 ± 0.16**	0.49 ± 0.24	**1.94 ± 0.28**	0.74 ± 0.11	**1.08 ± 0.21**	0.55 ± 0.11
***iniA***	0.26 **±** 0.05	0.05 ± 0.00	**1.41 ± 0.23**	**1.53 ± 0.30**	0.46 ± 0.02	**1.00 ± 0.00**	0.38 ± 0.18

The relative expression of the 11 genes was determined by comparison of the relative quantity of the respective mRNA in the presence of isoniazid at the MIC to the non-exposed condition. Each strain was assayed in triplicate using total RNA obtained from three independent cultures. Data was normalized to the *M*. *tuberculosis* 16S rDNA reference gene and presented as the mean fold change (±SD) compared with the control. Results in bold type were considered to be overexpressed. Relative expression levels above two were considered significantly overexpressed.

**Table 4:** Average quantification of the relative expression level,by RT-qPCR,of the genes that code for efflux pumps in *M. tuberculosis*exposed to rifampicin.

	Relative expression level ± SD				
Gene	82/09—MDR	149/09—XDR	286/09—XDR	69/11—MDR	29/12—XDR
***mmr***	**1.31 ± 0.62**	0.73 ±0.49	**2.49 ± 0.49**	**1.00 ± 0.33**	0.71 ± 0.00
***mmpL7***	**1.85 ± 0.87**	0.46 ±0.23	**3.23 ± 1.09**	0.39 ± 0.08	**1.02 ± 0.30**
***Rv1258c***	**1.40 ± 0.66**	**1.31 ± 0.44**	0.94 ± 0.18	0.20 ± 0.03	0.93 ± 0.31
***p55***	**4.27 ± 1.44**	**2.30 ± 0.23**	**9.88 ± 0.97**	**1.54 ± 0.66**	**2.59 ± 0.63**
***whiB7***	0.87 ± 0.29	**1.61 ± 0.76**	**1.04 ± 0.16**	**2.47 ± 0.24**	**1.83 ± 0.44**
***Rv1217c***	0.71 **±** 0.07	0.52 **±** 0.07	0.98 ± 0.76	0.14 ± 0.08	**1.12 ± 0.57**
***Rv1218c***	0.72 **±** 0.40	0.36 ± 0.12	0.69 ± 0.10	0.51 ± 0.05	**1.44 ± 0.62**
***efpA***	**1.94 ± 0.09**	**1.83 ± 0.44**	**1.41 ± 0.47**	**1.81 ± 0.27**	**3.47 ± 1.17**
***Rv2459***	0.69 ± 0.35	0.41 ± 0.04	0.74 ± 0.04	0.28 ± 0.08	**1.08 ± 0.21**
***pstB***	0.41 ± 0.30	0.46 ± 0.11	0.47 ± 0.04	0.22 ± 0.01	**1.41 ± 0.47**
***iniA***	**1.94 ± 0.28**	0.89 ± 0.25	**54.50 ± 21.35**	**1.47 ± 0.21**	**3.11 ± 1.20**

The relative expression of the 11 genes was determined by comparison of the relative quantity of the respective mRNA in the presence of rifampicin at the MIC to the non-exposed condition. Each strain was assayed in triplicate using total RNA obtained from three independent cultures. Data was normalized to the *M*. *tuberculosis* 16S rDNA reference gene and presented as the mean fold change (±SD) compared with the control. Results in bold type were considered to be overexpressed. Relative expression levels above two were considered significantly overexpressed.

**Fig 1:**
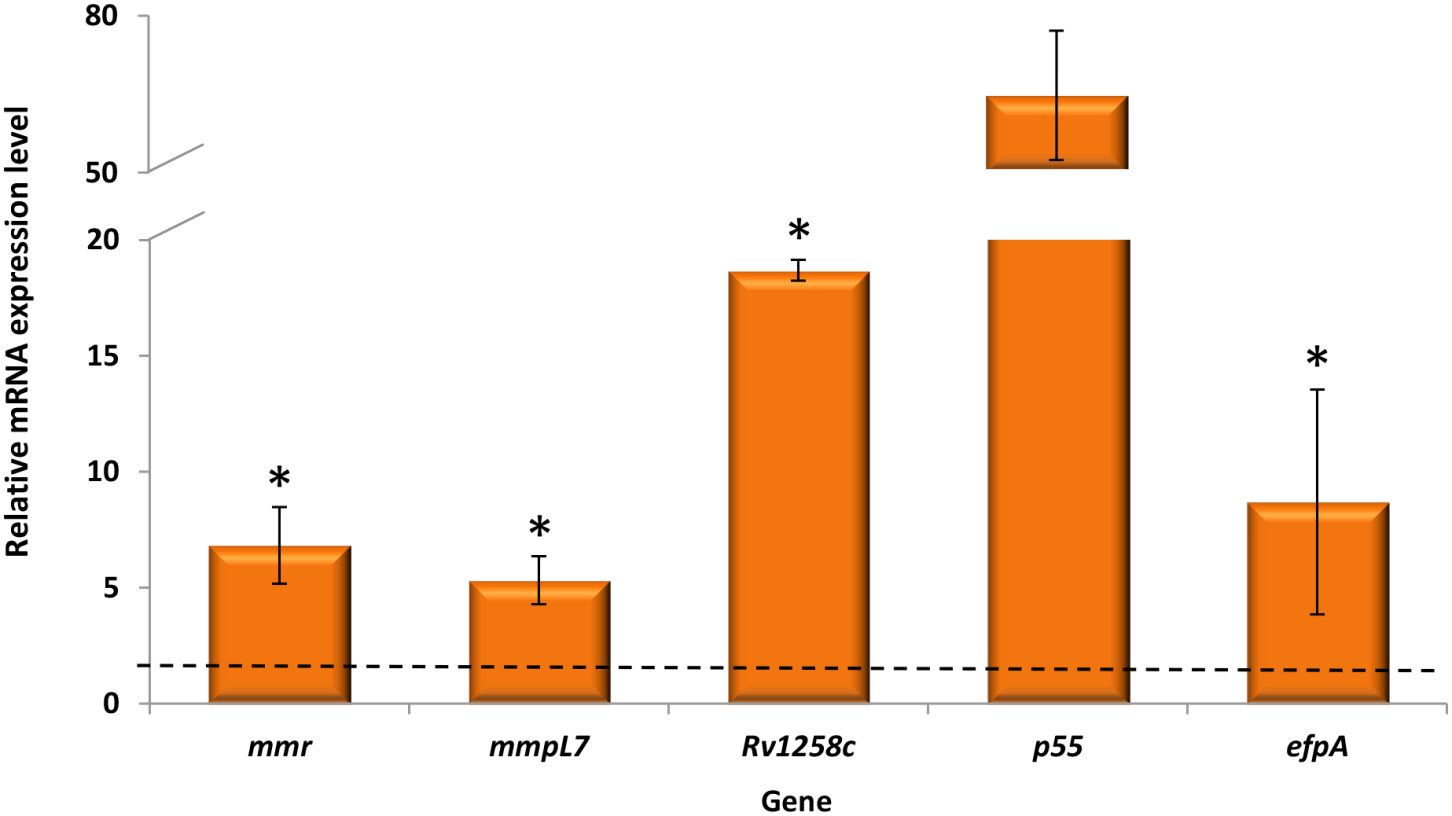
Analysis of intracellular *M. tuberculosis*efflux pump gene expression. Bacterial efflux pump gene expression was analysed after the intracellular growth of *M*. *tuberculosis* strain 82/09 exposed to isoniazid at 0.1 μg/ml during three days. After this period, macrophages were lysed and RNA extracted from macrophage-grown bacilli using a GTC/Trizol based method. Relative expression of the five genes was determined by comparison of the relative quantity of the respective mRNA in the presence of isoniazid to the untreated control using the 2^-ΔΔCt^ method. Data was normalized to the *M*. *tuberculosis* 16S rDNA reference gene and presented as the mean fold change (±SD) compared with the control (non-treated macrophage-grown bacilli). A level of relative expression equal to 1 (black dotted line in the graph) indicates that the expression level was identical to the non-exposed condition. Genes showing expression levels equal or above one, when compared with the non-exposed condition, were considered to be overexpressed. The assays were performed in triplicate. Results were considered significant when *P < 0.05.

Next, we sought to see if the expression of some of these pumps could be induced upon macrophage residence, *i*.*e*., in the macrophage harbouring the phagocytosed *M*. *tuberculosis*. As proof of evidence, we selected one MDR strain, *M*. *tuberculosis* 82/09, to study the expression of five efflux genes, *mmr*, *mmpL7*, *Rv1258c*, *p55* and *efpA*, and determined their expression level upon exposure to isoniazid inside macrophages. The results obtained show that indeed *M*. *tuberculosis* efflux pumps are also overexpressed inside macrophages upon exposure to isoniazid (Fig 1).

### Inhibition of ethidium bromide efflux demonstrates the role of the ion channel blockers as efflux inhibitors

**Fig 2:**
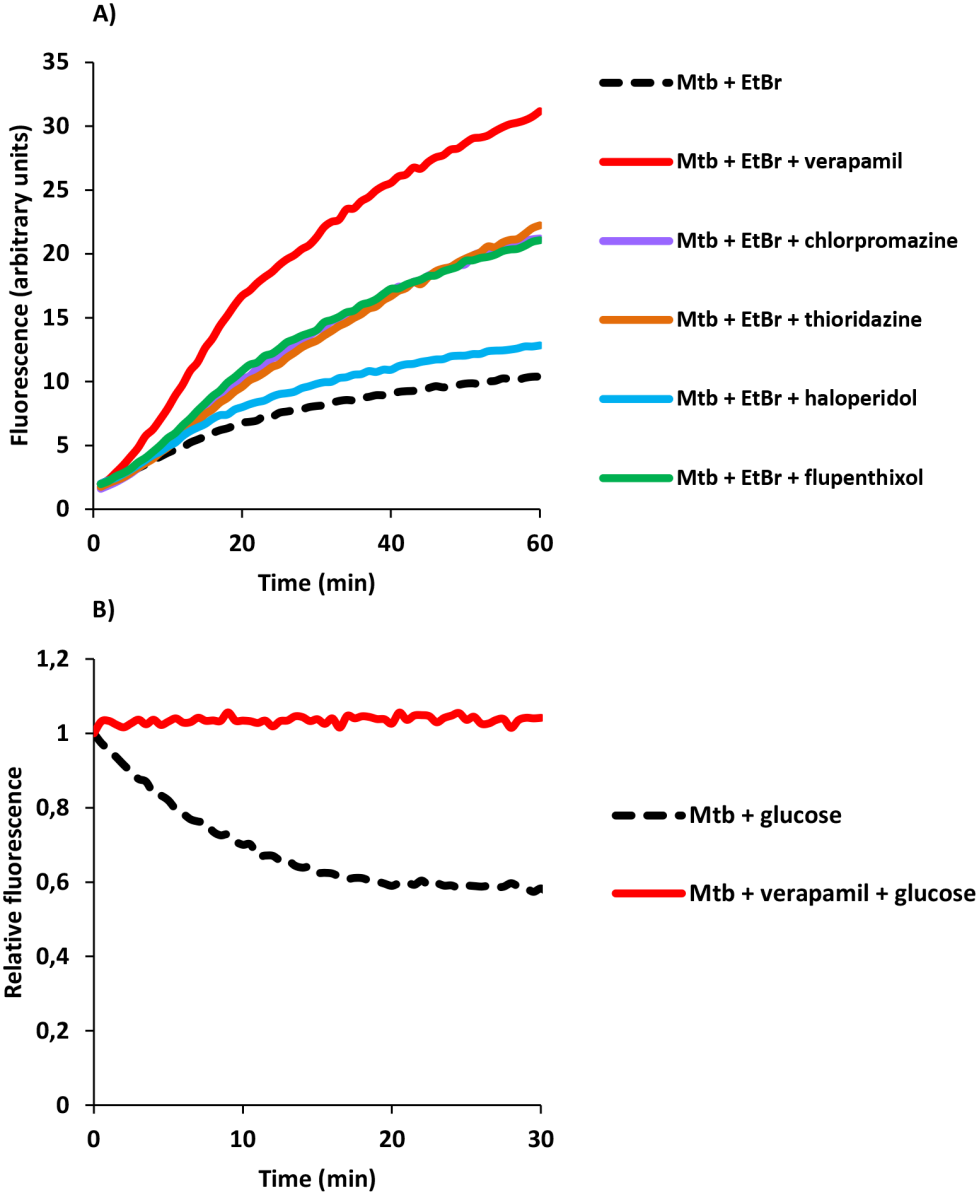
Effect of the inhibitors on the accumulation and efflux of ethidium bromide by the *M. tuberculosis*strains. In the figure is presented an assay for strain *M*. *tuberculosis* 29/12 as an example. A) Accumulation and B) efflux of ethidium bromide. Ethidium bromide was tested at 0.25 μg/ml. Efflux inhibitors were tested at half MIC. EtBr, ethidium bromide. Accumulation assays: results were considered significant when *P < 0.05 (EtBr plus flupenthixol or thioridazine) and highly significant **P < 0.01 (verapamil and chlorpromazine). Efflux assays: **P < 0.01.

To confirm the ability of the efflux inhibitors to interfere, on a real-time basis, with the efflux activity in *M*. *tuberculosis* inferred from the results previously obtained, efflux of ethidium bromide (a known bacterial efflux substrate) was evaluated for all strains by a fluorometric assay [3;19]. The effect of each compound on ethidium bromide accumulation by the *M*. *tuberculosis* strains are presented in Table 5 and a representative experiment is shown on Fig 2A. The relative final fluorescence (RFF) index corresponds to a measure of how efficient is the inhibitory effect of the compounds on efflux by comparison of the final fluorescence of the bacterial cells exposed to ethidium bromide in presence of the inhibitor against bacterial cells exposed only to ethidium bromide (taken as 0). The accumulation of ethidium bromide increased in the presence of all inhibitors. Verapamil promoted the highest increase in ethidium bromide accumulation in all strains, followed by flupenthixol (4/8), thioridazine (3/8) and chlorpromazine (2/8). Haloperidol was the compound that demonstrated the lowest inhibitory effect. Comparing between different strains, the MDR and XDR strains showed higher levels of ethidium bromide accumulation in presence of the inhibitors, whereas the clinical isoniazid monoresistant strains accumulated lower levels of ethidium bromide. To directly assess the efflux inhibitory activity of the compounds on these strains, we performed ethidium bromide efflux assays. Fig 2B shows a representative experiment in the presence of verapamil. Overall, the results showed that ethidium bromide efflux is considerably inhibited by the five compounds tested in the panel of *M*. *tuberculosis* strains studied.

**Table 5:** Relative final fluorescence (RFF) based on the accumulation of ethidium bromide for the *M. tuberculosis* strains in the presence of the inhibitors.

	RFF of the inhibitors				
Strain	Verapamil	Thioridazine	Chlorpromazine	Flupenthixol	Haloperidol
**H37Rv—Susceptible**	**1.58 ± 0.09**	0.97 ± 0.21	0.84± 0.03	0.90 ± 0.05	0.32 ± 0.10
**H37Rv**_**ΔkatG**_**—INH**^**R**^	**1.82 ± 0.28**	0.90 ± 0.07	**1.07 ± 0.004**	**1.06 ± 0.11**	0.39 ± 0.01
**82/09—MDR**	**2.17 ± 0.01**	0.98 ± 0.02	0.67 ±0.04	**1.25 ±0.008**	0.25 ± 0.01
**149/09—XDR**	**2.31 ± 0.02**	**1.13 ± 0.07**	**1.16 ± 0.11**	**1.45 ± 0.23**	0.58 ± 0.17
**69/11—MDR**	**1.66 ± 0.06**	**1.17 ± 0.07**	0.47 ± 0.21	**1.44 ± 0.0005**	0.21 ± 0.006
**29/12—XDR**	**2.00 ± 0.21**	**1.14 ± 0.11**	**1.04 ± 0.03**	**1.03 ± 0.14**	0.24 ± 0.04
**294/09—INH**^**R**^	**1.21 ± 0.15**	0.52 ± 0.03	0.44 ±0.08	0.98 ±0.06	-0.18 ±0.02
**269/03—INH**^**R**^	**1.68 ± 0.24**	0.78 ± 0.02	0.45 ± 0.02	0.87 ± 0.15	0.21 ± 0.12

The effect of the inhibitors in the accumulation of ethidium bromide was interpreted as follows: RFF values above zero indicated that bacterial cells accumulate more ethidium bromide under the condition used than those of the control (non-treated bacterial cells). In case of negative RFF values, these indicated that treated bacterial cells accumulated less ethidium bromide than those of the control condition. Values in boldface (above 1) indicate enhanced accumulation of ethidium bromide in the presence of efflux inhibitors. Each assay was performed in triplicate and the results presented correspond to the average of three independent assays plus standard deviation (**±** SD). Strain 286/09 was not evaluated due to poor growth under the conditions required for these assays.

### The ion channel blockers display rapid and high bacterial killing activity associated with ATP depletion

**Fig 3:**
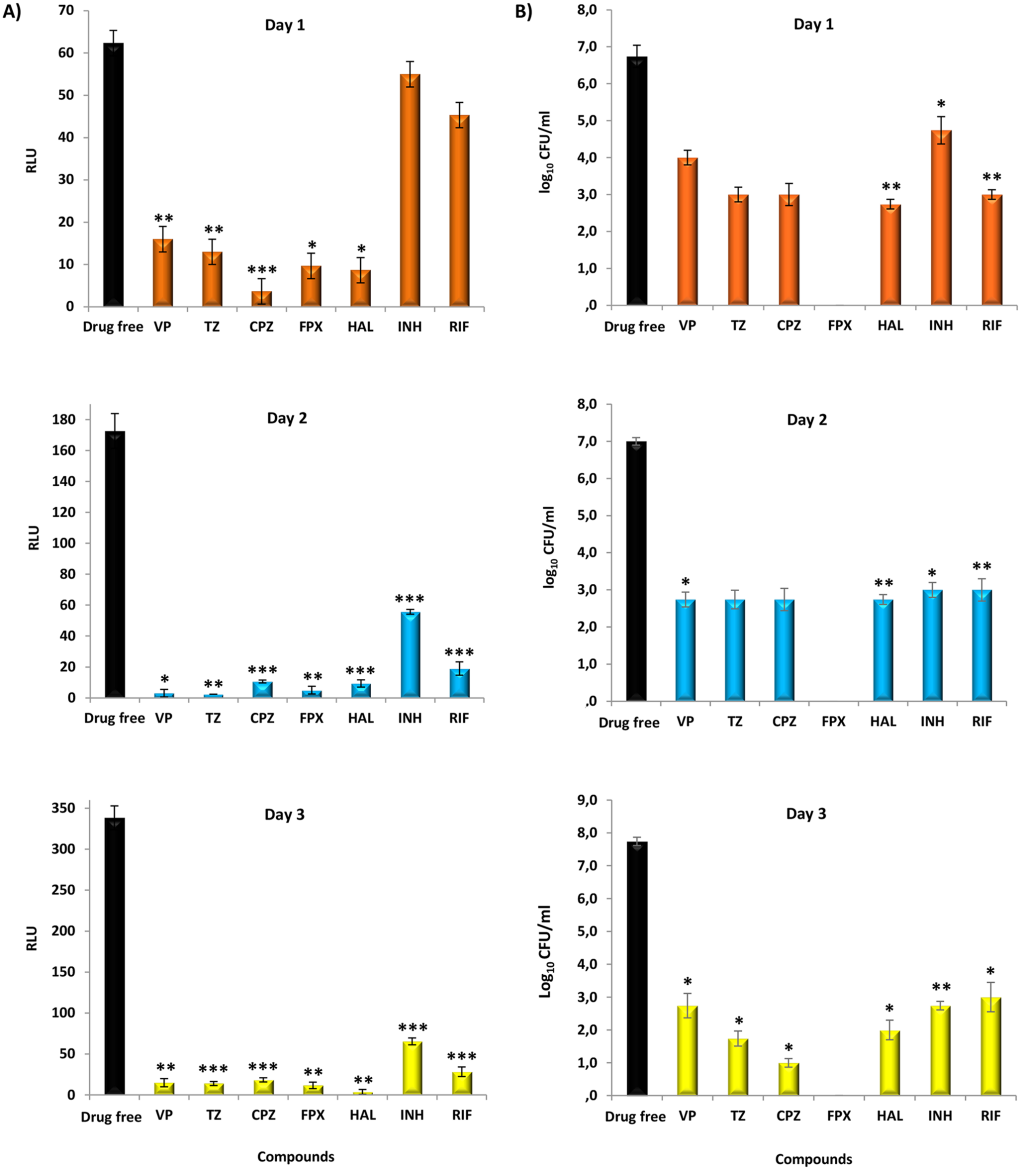
Mycobacterial intracellular ATP levels A) and viability B). M. tuberculosis H37Rv was exposed to the inhibitors (at 5X MIC) during three days. Bacterial viability was measured with MGIT960 system at day one, two, and three as described in Material and Methods. ATP was determined by using a luciferin-luciferase bioluminescence detection system at day one, two and three. Isoniazid (INH) and rifampicin (RIF) were used as controls. VP, verapamil; TZ, thioridazine; CPZ, chlorpromazine; FPX, flupenthixol; HAL, haloperidol. RLU, relative fluorescence units. The results were considered significant when *P < 0.05 and highly significant when **P < 0.01 and ***P <0.001.

Once demonstrated that extrusion of substrates by active efflux in *M*. *tuberculosis* increases tolerance towards antimycobacterial agents, which can be reduced/inhibited by the ion channel blockers studied, to determine whether the treatment with the ion channel blockers could have an effect on bacterial ATP production, we monitored the intracellular ATP levels over three days along with their bactericidal activity using standard time-kill assays against the reference strain H37Rv with all inhibitors and antibiotics at 5X MIC. These concentrations were chosen based on the fact that *in vitro* grown nonreplicating *M*. *tuberculosis* has reduced susceptibility to the antituberculosis drugs and are intended to exacerbate the mechanism of action of the compounds in order to be readily measured and compared [21; 24]. ATP levels and killing activity were recorded after one, two, and three days of exposure to the compounds and the results are shown in Fig 3A and 3B, respectively. A rapid drop in intracellular ATP levels of *M*. *tuberculosis* H37Rv was noticed after one day of exposure to all ion channel blockers, which progressively falls until the third day of exposure. This decrease in the ATP levels upon exposure to the ion channel blockers correlated with the reduction of bacterial viability (Fig 3A and 3B –upper panel). Conversely, the exposure to isoniazid or rifampicin had no significant effect on the ATP levels after one day of exposure (Fig 3A and 3B –upper panel) showing that the drop in ATP levels we observed on the first day of exposure is specific to the ion channel blockers. After the second day, bacterial viability and ATP content falls for all compounds in a non-specific manner (Fig 3A and 3B –middle and bottom panel). Of note, although no growth was obtained for H37Rv treated with flupenthixol (from day one on), ATP content was still detected reaching, at day three, similar levels than those observed at day 0 (data not shown). In order to not underestimate nonreplicating populations upon drug exposure we used a broth method to determine mycobacterial viability. In this case, we assume that the bacilli exposed to flupenthixol enter in a nonreplicating state after the first day of exposure. The ATP content seems not decline much after the stress caused by flupenthixol exposure when comparing with day 0 reflecting the presence of *M*. *tuberculosis* with reduced metabolism. *de novo* ATP synthesis (although at low levels) is necessary to maintain mycobacterial viability [24] which explains why we detected ATP even in the absence of growth. However, the exposure to all compounds eventually leads to mycobacteria death. The sterilizing effect of the compounds was shown by the lack of growth after a prolonged incubation period in media without the compound (see Material and Methods for details). These results make evident the ability of *M*. *tuberculosis* to survive under severe antimicrobial conditions and highlight the early-bactericidal effect promoted by the ion channel blockers, most probably due to the rapid energy depletion, a condition that is of utmost importance for their *in vivo* potential use as adjuvants of tuberculosis chemotherapy for treatment shortening [2].

### The ion channel blockers possess antibacterial activity against intracellular *M*. *tuberculosis*

**Fig 4:**
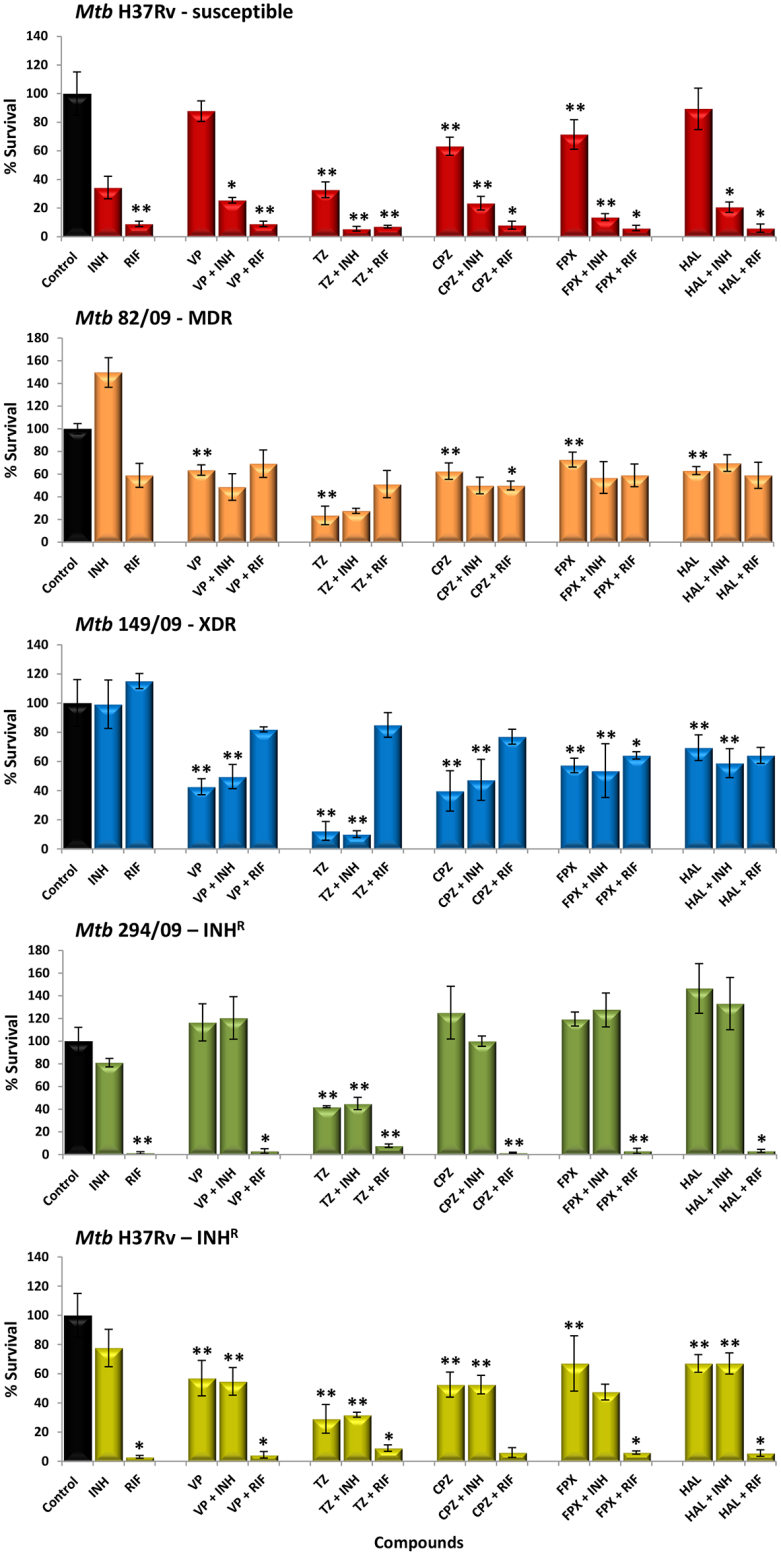
Antimycobacterial activity of the ion channel blockers against *M. tuberculosis*-infected macrophages. Effect of the inhibitors on the intracellular survival of *M*. *tuberculosis* (Mtb) within human monocyte-derived macrophages, three days post infection. Isoniazid (INH) was tested at 0.1 μg/ml, verapamil (VP), 10 μg/ml; thioridazine (TZ), 2.5 μg/ml; chlorpromazine (CPZ), 1.25 μg/ml; haloperidol (HAL), 1.25 μg/ml; flupenthixol (FPX), 1.25 μg/ml. The results are presented as a mean of the percentage of the survival ± SD. RIF, rifampicin; R, resistant; MDR, multidrug resistant; XDR, extensively drug resistant. The results were considered significant when *P < 0.05 and highly significant when **P < 0.01.

To assess the ability of the ion channel blockers to potentiate the bactericidal activity of isoniazid and rifampicin within macrophages, the following experiments were carried out in human monocyte-derived macrophages: a) determination of the toxicity levels of the ion channel blockers to guarantee nontoxic concentrations to be used in the macrophage experiments, and b) infection assays with H37Rv strain and four drug resistant strains, with commonly described resistance-associated mutations in *katG*, *inhA*, and *rpoB* (one MDR, one XDR and two isoniazid monoresistant strains), followed by treatment with these compounds. The toxicity levels of the compounds are presented in S1 Fig. The antimycobacterial activity of the compounds in combination with isoniazid or rifampicin within macrophages is presented in Fig 4. The synergistic effect of each compound alone or in combination with isoniazid or rifampicin was compared to the control macrophages with no treatment. All compounds impaired growth of either wild-type or drug resistant intracellular *M*. *tuberculosis*, with thioridazine showing the highest effect, with an average of intracellular killing of mycobacteria of 58% to 88%. The remaining compounds increase the macrophage rates of killing by 12% to 64% (verapamil), 37% to 60% (chlorpromazine), 27% to 43% (flupenthixol) and 11% to 37% (haloperidol). For strain 294/09, only thioridazine was able to reduce the CFU number.

The co-administration of isoniazid with any of the compounds only resulted in strong potentiation of the killing effect for strain H37Rv. No additional killing by co-administration of isoniazid (at the critical concentration of 0.1 μg/ml) was observed for the isoniazid-resistant strains since all strains, except H37Rv, harboured a mutation that confers isoniazid resistance. In respect to rifampicin, similar results were obtained (Fig 4). The combination between the inhibitors and rifampicin reduced the number of viable bacteria by 91% to 98% on the rifampicin-susceptible strains H37Rv, H37Rv_ΔkatG_ and 294/09. In respect to the rifampicin-resistant strains, the combination of rifampicin with an ion channel blocker reduced the CFUs by 31% to 50% for strain 82/09 and 15% to 36% for strain 149/09. Of note, the combination of thioridazine and rifampicin can result into an antagonistic effect for strain 149/09 (XDR). It is known that rifampicin resistant strains have increased fitness in the presence of rifampicin [25]. It is possible that the reduced concentration of rifampicin we used in these assays (compared with the rifampicin MIC for this strain) is promoting bacterial replication. Additional studies are necessary to address this issue. Together, these results show that thioridazine, verapamil, chlorpromazine, flupenthixol, and haloperidol strengthen the macrophage killing activity against intracellular *M*. *tuberculosis* irrespective of its resistance pattern at clinical relevant concentrations and that are well tolerated by the human macrophage.

### The ion channel blockers promote vacuolar acidification

The transport of cations by eukaryotic vacuolar efflux pumps may control conditions in the vacuole that determine the effectiveness of the microbial killing and digestion via lysosomal hydrolytic enzymes, coupled to the energy provided by membrane embedded electron transport chain enzymes and proton channels [26; 27]. One possible explanation for these observations is the concentration of these inhibitors by the phagosome, a known physiological phenomenon demonstrated by pharmacodynamic studies of these compounds for clinical usage [28]. This results in intracellular concentrations that will increase the transcription of the proton pump v-ATPase’s, promoting phagosomal acidification [29]. This will increase the concentration of Ca^2+^ in the lumen [30], forcing the acidification-dependent activation of lysosomal hydrolases [29] and subsequent enhancement of the macrophage-mediated killing of the mycobacteria [31; 32].

**Fig 5:**
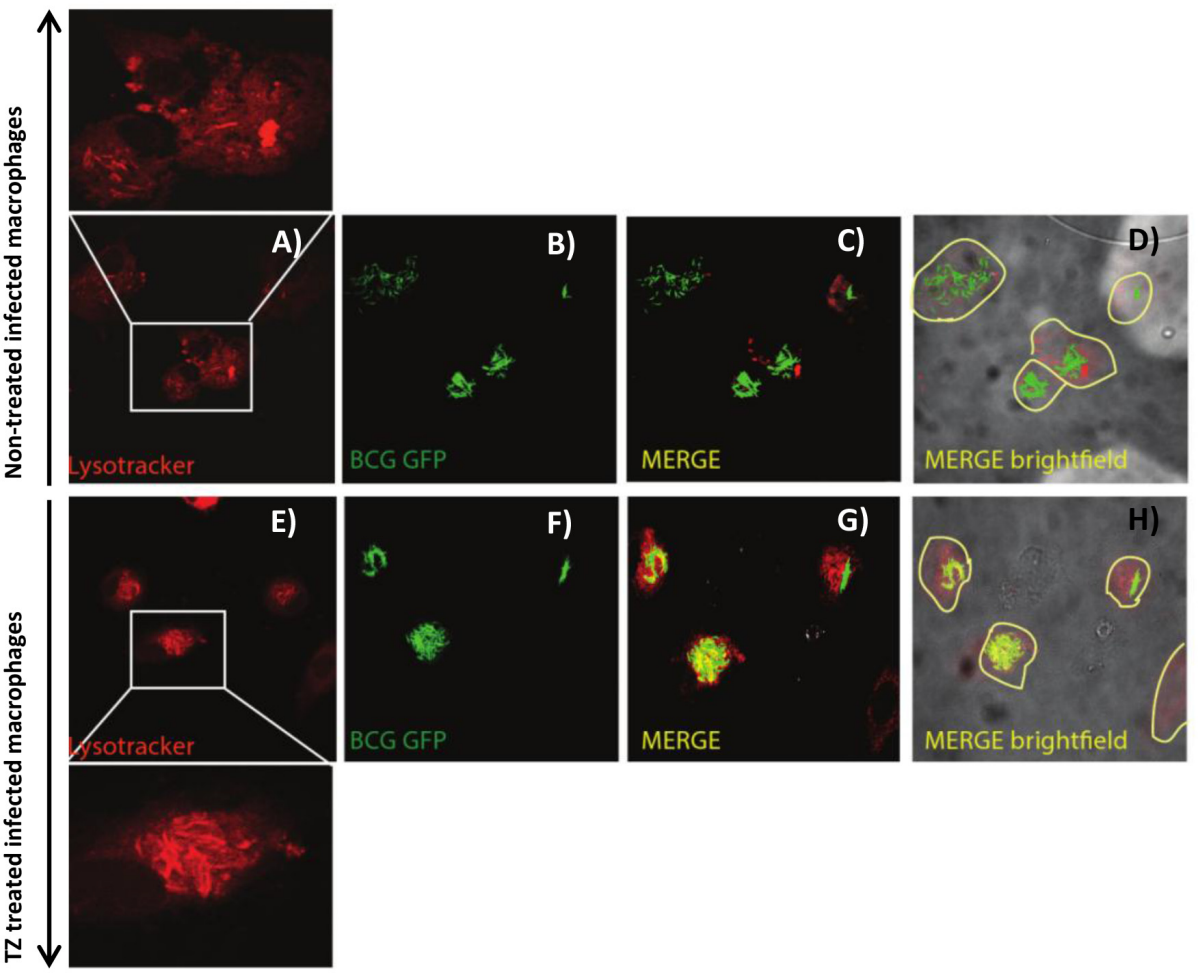
Effect of thioridazine on endocytic vesicles/phagosomes acidification. Confocal microscopy of human monocyte-derived macrophages infected with *M*. *bovis* BCG-GFP and stained with LysoTracker Red. A) to C): non-treated cells and (D) brightfield for non-treated cells; E) to G): cells treated with 2.5 μg/ml thioridazine (TZ) and (H) brightfield for cells treated with 2.5 μg/ml thioridazine. Comparing the non-treated cells with the treated ones, the LysoTracker staining is noticeable after macrophage treatment with the compounds. We can also observe co-localization of *M*. *bovis* BCG-GFP with LysoTracker Red. Data was analysed using a LSM 510 META laser scanning confocal microscope.

**Fig 6:**
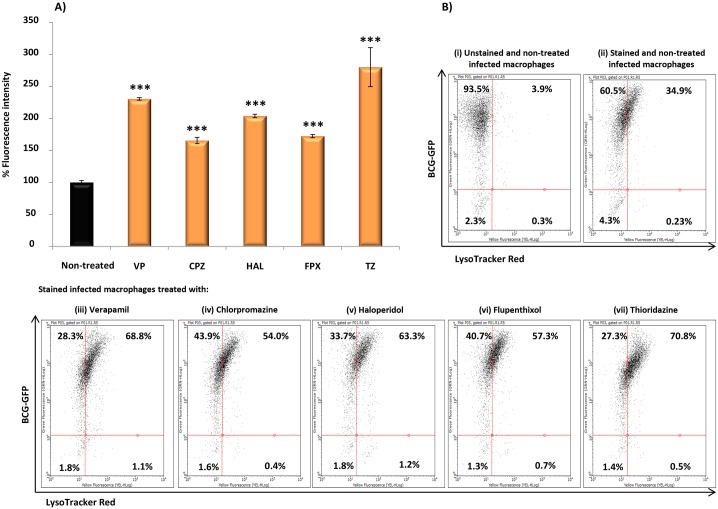
Relative quantification of phagosomal acidification in *M. bovis* BCG-GFP-infected macrophages stained with LysoTracker Red and analysed by flow cytometry. Human macrophages were infected with *M*. *bovis* BCG-GFP, treated with verapamil (VP), thioridazine (TZ), chlorpromazine (CPZ), flupenthixol (FPX), and haloperidol (HAL). Data was analysed by flow cytometry using an easyCyte^™^ 5HT flow cytometer. A) Bars graph: quantification of the increase on the overall number of LysoTracker Red stained vesicles per cell (average fluorescence intensity); B) cytograms for (i), unstained and non-treated infected macrophages; (ii), stained and non-treated infected macrophages; and (iii) to (vii), stained and infected macrophages treated with VP, TZ, CPZ, FPX and HAL. VP was tested at 10 μg/ml; TZ at 2.5 μg/ml; CPZ, HAL and FPX at 1.25 μg/ml. Significance of the results was tested using Student’s t-test and were considered highly significant, ***P <0.001.

**Fig 7:**
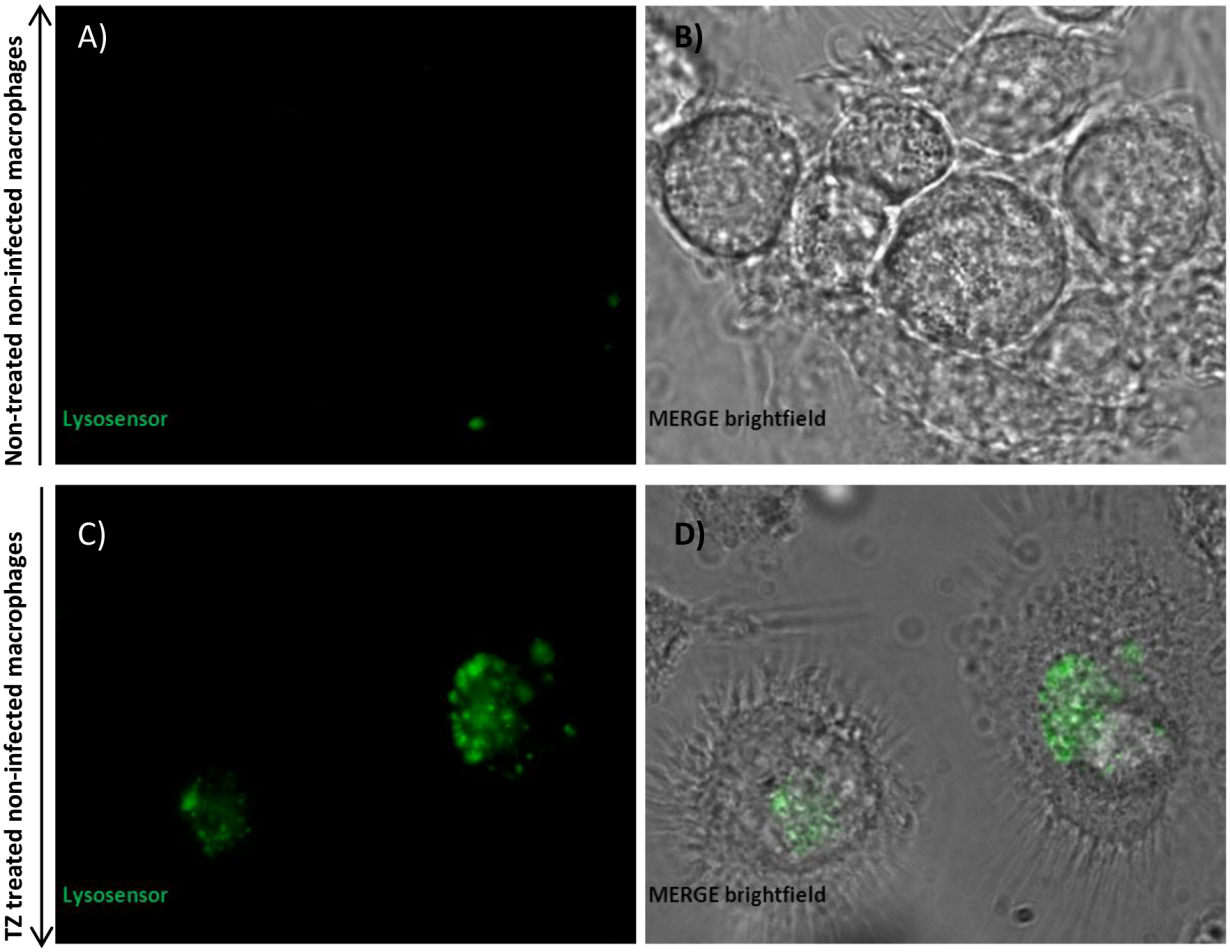
Qualitative analysis of phagosomal acidification in macrophages stained with LysoSensor Yellow/Blue by confocal microscopy. Evaluation of fluorescence intensity of (A) non-infected non-treated macrophages and (B) brightfield for non-infected non-treated macrophages; evaluation of fluorescence intensity of (C) non-infected macrophages treated with thioridazine (TZ) and (D) brightfield for non-infected macrophages treated with thioridazine, with the aid of the fluorescent probe LysoSensor Yellow/Blue. LysoSensor Yellow/Blue DN160 emits yellow/dark green fluorescence at neutral pH and blue/green fluorescence at pH 5–5.5. Data was analysed using a LSM 510 META laser scanning confocal microscope.

**Fig 8:**
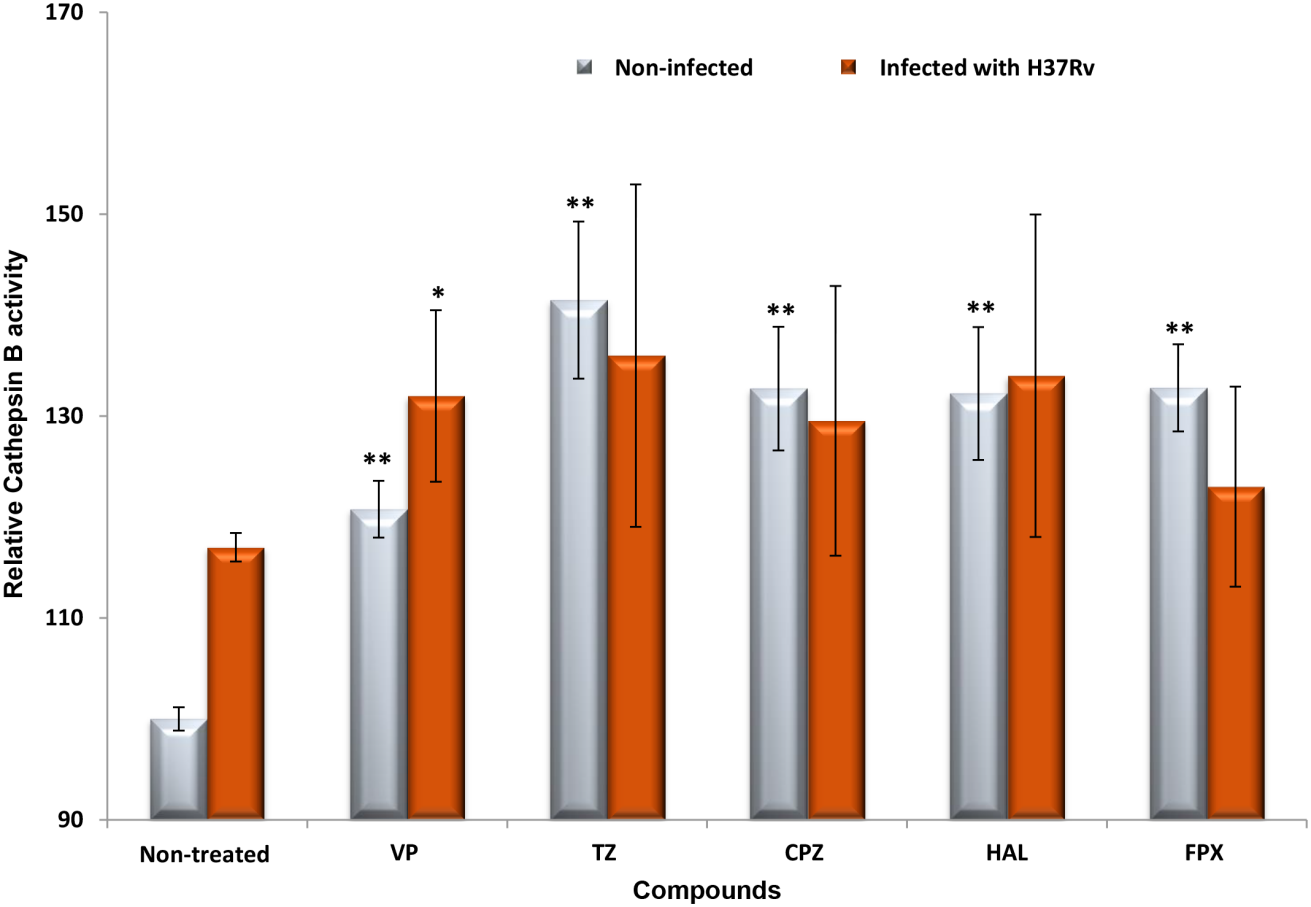
Determination of cathepsin B activity. Cathepsin B activity was evaluated in human monocyte-derived macrophages infected and non-infected with *M*. *tuberculosis* H37Rv and non-treated and treated with verapamil (VP), thioridazine (TZ), chlorpromazine (CPZ), flupenthixol (FPX) and haloperidol (HAL). VP was tested at 10 μg/ml; TZ at 2.5 μg/ml; CPZ at 1.25 μg/ml; HAL at 1.25 μg/ml; FPX at 1.25 μg/ml. The results were considered highly significant, **P < 0.01.

To test this hypothesis, the effect of these compounds on vacuolar acidification was tested against human monocyte-derived macrophages. As “proof of concept” we choose *M*. *bovis* BCG as model of infection since *M*. *bovis* BCG can block phagosome acidification [23]. Additionally, the requirement of live cells by the LysoSensor method, together with biosafety restrictions led us to use this model of infection. Macrophages were treated with the compounds and stained with LysoTracker and/or LysoSensor, both acidotropic probes. Initially, *M*. *bovis* BCG-infected macrophages were treated with the compounds and stained with LysoTracker Red. As shown in Fig 5, comparing the non-treated infected macrophages (A to D) with the treated ones (E to H), the LysoTracker staining increased after macrophage treatment with the compounds, indicating that they induce an increase of the number of acidic endosomal vesicles in the infected macrophages treated with the compounds. Also, in infected macrophages, we observed co-localization of *M*. *bovis* BCG with LysoTracker Red (C-D and G-H), indicating that the compounds mediate acidification of *M*. *bovis* BCG-containing vesicles. To further quantify the extent of macrophage vesicle acidification we measured the accumulation of LysoTracker Red in infected macrophages, using flow cytometry (Fig 6). Non-treated *M*. *bovis* BCG-infected macrophages increase phagosome acidification by approx. 31%. After treatment with the compounds, a significant increase in the percentage of cells stained positive for LysoTracker Red was noted (Fig 6B—cytogram). The increased fluorescence in the treated macrophages that incorporates both *M*. *bovis*-GFP and LysoTracker RED ranges from 23% for chlorpromazine, 26.4% for flupenthixol, 32.3% for haloperidol, 37.8% for verapamil, to 39.8% with thioridazine. Next we measured the increase on the overall number of LysoTracker Red stained vesicles per cell (average fluorescence intensity) and observed that all the compounds increased the overall number of stained vesicles when compared with the non-treated infected macrophages (Fig 6A–bars graph). Overall, verapamil, thioridazine and haloperidol induced the strongest effects. After demonstrating that all these compounds increase phagosome acidification we analysed the fluorescence intensity of the non-infected macrophages non-treated (Fig 7A and 7B) and treated (Fig 7C and 7D) with the compounds with the aid of the fluorescent probe Lysosensor Yellow/Blue. LysoSensor Yellow/Blue emits yellow/green fluorescence at neutral pH and blue/green fluorescence at pH 5–5.5. The green fluorescence observed in treated macrophages (Fig 7) indicates that the pH of the vesicles is around 5–5.5. Additionally, in *M*. *bovis* BCG-GFP-infected macrophages treated with the compounds and stained with LysoSensor, we also observed co-localization of *M*. *bovis* BCG-GFP within acidic vesicles (data not shown). Collectively, these results allow us to show that the ion channel blockers are able to induce the acidification of endocytic vesicles independently if the macrophage is infected or not. *M*. *tuberculosis* is able to survive to some extent in the acidic environment of the phagolysosome of activated macrophages and its survival or elimination depends on the ability of the macrophage to activate its pH dependent lysosomal hydrolases [33]. To test this hypothesis with these compounds and explain the enhanced killing activity described above, we measured the activity of cathepsin B, a lysosomal cysteine protease. Increased activity of cathepsin B was detected in cultures treated with all the compounds (Fig 8) regardless whether the macrophage is infected or not. These observations indicate that the inhibitors prompted the acidification of the phagosomes, including the ones containing *M*. *tuberculosis*, and enhanced the proteolytic activity of cathepsin B promoting the acidic hydrolytic features of the phagosome, thus providing further evidences of their physiological effects on the macrophages.

## Discussion

The global emergence of MDR and XDRTB has hampered the perspectives of tuberculosis control and elimination. For this reason there is an urgent need for the identification of compounds with different mechanisms of action that can be used in combination with the existing antituberculosis drugs to shorten the treatment. We have previously observed that ion channel blockers can increase the efficacy of antibiotics, *in vitro*, due to their ability to inhibit efflux pump activity, and that they also increase the killing activity of non-killing macrophages against intracellular *M*. *tuberculosis* [3; 9; 12; 31; 32]. These observations raised the question of how these inhibitors enhance the killing of *M*. *tuberculosis*. We have hypothesized that the treatment with ion channel blockers restricts *M*. *tuberculosis* growth by interference with bacterial metabolism as well as enhance the killing activity of the macrophage. To this end, we investigated the activity of a group of compounds, which share the common property of inhibiting ion channels, against MDR and XDR *M*. *tuberculosis* clinical strains *in vitro* and in macrophages. Here we show that (i) the ion channel blockers are effective against MDR and XDR *M*. *tuberculosis* strains *in vitro* and in human macrophages, (ii) the bactericidal action of the ion channel blockers against *M*. *tuberculosis* strains is associated with their interference with bacterial energy metabolism; and (iii) they increase phagosome acidification and the expression of phagosomal hydrolases.

First we tested the synergistic activity of the several ion channel blockers in combination with the most effective antituberculosis drugs, isoniazid and rifampicin, and the second-line drugs amikacin and ofloxacin, on *M*. *tuberculosis* strains with different resistance patterns. The compounds were able to potentiate the activity of the first-line antibiotics, in either drug susceptible or resistant strains, reducing the resistance levels from high- to low-level despite the presence of mutations conferring resistance. Regarding isoniazid, the effect of these compounds seems to be limited to strains with mutations in *inhA* gene as little or no effect was observed in the strains harbouring a mutation in the *katG* gene. This result is in agreement with the fact that mutations in the *katG* gene lead to high-level resistance [13]. Significant reduction of the rifampicin resistance levels were noted for the MDR strains in the presence of verapamil and chlorpromazine. For all five rifampicin resistant strains the resistance was reduced from 320 μg/ml to 20 μg/ml (Table 2). These results further indicate that rifampicin is able to induce efflux pump activity in rifampicin resistant *M*. *tuberculosis* strains, as reported by others [8]. Fluoroquinolones are known efflux pump substrates in non-mycobacterial species [34] and some reports allege that efflux play an important role in the development of fluoroquinolone resistance in *M*. *tuberculosis* [35]. In the present study, no reduction in the ofloxacin resistance levels was observed for the three ofloxacin resistant clinical strains carrying mutations in the *gyrA* gene, indicating that efflux has little contribution to the resistance in these XDR *M*. *tuberculosis* strains. Likewise, for amikacin, no effect was observed for two out of the three amikacin resistant strains studied, indicating that the presence of a mutation in *rrs* is the solely cause of resistance in these strains. So far, only resistance to streptomycin has been associated with efflux activity in *M*. *tuberculosis* [36; 37], with no reference to amikacin. It is possible that the different chemical structures make amikacin a poor substrate of efflux, as suggested by our study, and further studies are necessary to assess the contribution of efflux to aminoglycosides resistance in *M*. *tuberculosis*. Interestingly, resistance to amikacin was reversed in the clinically amikacin high-level resistant strain 69/11. This strain was shown to harbour a heteroresistant population towards amikacin *i*.*e*. with demonstrable coexistence of populations of bacilli susceptible and resistant to amikacin. This is particularly important from the clinical point of view, since heteroresistance has been implicated in the development of antibiotic resistance where the overexpression of efflux pumps plays an important role in guaranteeing the survival of a low-level resistant population until genetic determinant for resistance (mutation) occurs and becomes established in the population, assuring high-level resistance [3; 4; 8]. The fact that strains changed their resistance profile from high- to low-level upon the addition of some of the inhibitors can bring clinical implications. In the presence of a high-level resistant strain, these drugs are predicted to be useless. Although the degree of synergism does not reach full susceptibility the reduction of the MIC value of the antibiotics to values close or within the serum concentration can improve therapy outcomes. Additionally, it is known that antibiotics can penetrate and accumulate in phagocytic cells [38] thereby increasing the therapeutic concentrations. The effectiveness of the synergistic combination of these inhibitors with conventional therapy, seen *in vitro*, is expected to be beneficial in reducing the antibiotics resistance levels resulting in more effective therapy against *M*. *tuberculosis* drug resistant strains.

To verify the connection between the resistance, efflux activity and the ability of these compounds to inhibit efflux pump activity in these drug resistant strains, real-time fluorometry, using ethidium bromide as fluorophore, was used in the presence and absence of the compounds in evaluation. When the efflux activity of the drug resistant clinical strains was compared with that of the reference strain H37Rv, all strains showed higher efflux capacity, which could be inhibited in the presence of subinhibitory concentrations of all the five compounds tested. Among these, verapamil was the one that showed the most potent effect in the real-time inhibition of the efflux of ethidium bromide, followed by flupenthixol, thioridazine, chlorpromazine, and only marginally by haloperidol. Furthermore, the contribution of the *M*. *tuberculosis* membrane transporters for this increased efflux activity and resistance towards isoniazid and rifampicin was confirmed by a general and marked overexpression of almost all efflux genes tested by RT-qPCR. Although we cannot observe a specific pattern of expression, the genes *mmr*, *mmpL7*, *p55*, *Rv1217c* and *efpA* showed a significant increase in their expression levels in the presence of isoniazid. Regarding the exposure to rifampicin, we observed an increase in the expression of the majority of the genes albeit at lower absolute levels than those obtained for isoniazid. Of all pumps studied, P55 a member of the major facilitator superfamily of efflux transporters, described by da Silva et al [39] was consistently expressed in all strains, presenting a 1.54 to 9.88-fold increase in the presence of rifampicin. The synergistic effect of the efflux inhibitors seems to be dependent on the strains and this is an expected result once we accept the contribution of efflux to the overall drug resistance level. The resistant level of a given strain has an efflux component and the other is related to the mutation in a drug resistance target. With the concentrations of the efflux inhibitors we have tested we can only aim to reduce the efflux component but the resistance component due to the mutation remains untouched. Overexpression of efflux pumps was more evident on multidrug resistant strains than in isoniazid monoresistant strains and the compounds shown to be more effective against the multidrug resistant strains overexpressing efflux pumps. Monoresistant strains have little efflux capacity as can be observed in Table 5. These results indicate an association between drug resistance, the genetic background, the pattern of overexpression of efflux pumps and consequent efflux ability which in turn influence the efficacy of the compounds at the concentrations necessary to act as efflux inhibitors.

A second important contribution of the current study is our finding that efflux pumps of multidrug resistant *M*. *tuberculosis* clinical strains are overexpressed inside the macrophages upon drug exposure, mimicking the inducible adaptative response seen *in vitro*. Adams et al [11; 40], using the zebrafish larval model, demonstrated that drug-tolerant bacteria originate in macrophages by the inducement of bacterial efflux pumps, which rendered the bacteria tolerant to several antituberculosis drugs. Collectively, these results reinforce the concept that induction of efflux pumps is one of the mechanisms involved in the acquisition and maintenance of drug resistance in *M*. *tuberculosis*. Our previous observations with laboratory induced-isoniazid resistant *M*. *tuberculosis* strains showed that efflux pumps in *M*. *tuberculosis* are promiscuous in their activity as we could not unequivocally associate extrusion of isoniazid [3]. The present study allowed us to observe the same kind of effect on *M*. *tuberculosis* multi and extensively drug resistant clinical strains in respect to isoniazid resistance which can be extended to rifampicin resistance. Induction of efflux pumps seems to be a general stress response to the presence of the antibiotics and the differences observed in gene expression profiles in clinical strains most probably reflect their history of different antibiotic pressures in the clinical setting, alongside with their genetic backgrounds.

To unravel the general bactericidal mechanism of action of the five ion channel blockers, time-kill studies were carried out to assess the rate of killing caused by the compounds in parallel with the determination of ATP levels. All compounds were found to quickly deplete intracellular ATP which was accompanied by a rapid and high bactericidal effect against both drug susceptible and drug resistant *M*. *tuberculosis* strains, corroborating the studies of Warman et al [21] and de Knegt et al [41]. The demonstration that the early bactericidal effect promoted by the ion channel blockers is due to their direct interference on the bacterial cell energy, as previously hypothesized [42], was further supported by the absence of such response following exposure to the antibiotics isoniazid and rifampicin at the same antimicrobial conditions (Fig 3). These results confirm that ATP depletion occurs as a consequence of exposure to the ion channel blockers and indicates that these compounds directly affect the metabolic state of the bacteria with anticipated effect on available bacterial energy for active efflux. Several other drugs, whose mechanism of action is known to affect the mycobacterial metabolic energy and respiration, are now in the pipeline or already in clinical use against MDR and XDRTB, such as bedaquiline, an ATP synthase inhibitor [43] and PA-824 plus delamanid that target the cytochrome oxidase [44]. Moreover, it was recently demonstrated that bedaquiline is able to inhibit active efflux in *Mycobacterium smegmatis* [45]. Prokaryotic and eukaryotic efflux pumps use energy of either ATP (the primary transporters) or PMF (secondary transporters) for the extrusion of ions, metabolites or noxious compounds using oxidative phosphorylation as their main source of energy [42; 46]. This chain of energy production starts in the prokaryotic or eukaryotic NADH dehydrogenase and ends up at the respective F_1_F_0_-ATP synthase, key electron transport chain enzymes that have been hypothesized to be directly or indirectly inhibited by ion channel blockers [42; 46; 47]. To our knowledge, the results obtained here are the first reported evidences that the bactericidal action of the ion channel blockers against *M*. *tuberculosis* clinical strains is associated with their interference with energy metabolism.

The use a macrophage model allowed us to further assess the intracellular activity of the ion channel blockers against susceptible and resistant *M*. *tuberculosis* strains, in order to evaluate the dual prokaryotic-eukaryotic properties of these compounds. We observed that all these compounds lead to a significant decrease in the intracellular mycobacterial load as result of increased phagosome acidification and enhanced activation of lysosomal hydrolases. Our previous research has leaded us to propose that the enhancement of the killing activity of macrophages by this type of compounds was dependent upon the availability of potassium and calcium ions inside the cells [12; 31; 32]. In support of these results, Gupta et al [30] elegantly demonstrated that the inhibition of L-type ion channels results in significant increase of calcium from intracellular stores within macrophages leading to a reduction in the mycobacterial burden. More recent studies with imatinib, an inhibitor of the Abelson tyrosine kinase and also a calcium channel blocker, used for the treatment of cancer and pulmonary arterial hypertension, showed its inducement of phagosomal acidification and growth restriction of *M*. *tuberculosis* in human macrophages [29]. Here we demonstrated that phagosome acidification occurs as a direct effect of the ion channel blockers, independently if the macrophage is infected or not. This means that these compounds can restrict *M*. *tuberculosis* growth independently of the phenotype of the infecting strain. Altogether, these results may afford a new strategy for the design and optimization of new compounds towards intracellular *M*. *tuberculosis*.

These compounds have been used for years as antipsychotics and antihypertensives, are affordable and, can be taken as oral formulation. Nevertheless, although haloperidol, thioridazine and chlorpromazine have been widely used for the treatment of psychosis, these compounds have significant toxic effects on mitochondrial bioenergetics function which may result in serious tardive dyskinesia, Parkinson-like disease [48; 49] and cardiotoxicity [50]. Conversely, when administered orally verapamil is extremely well tolerated with few side effects documented [51]. The significant differences observed in the effective concentrations of the ion channel blockers required to inhibit *M*. *tuberculosis in vitro* versus those needed to produce similar effects on macrophages (S3 Table), possibly indicates the ability of the macrophages to concentrate each compound. Here we have demonstrated the activity of verapamil, thioridazine, chlorpromazine, flupenthixol and haloperidol against intracellular drug resistant *M*. *tuberculosis* at nontoxic concentrations, usually reached in the human serum, when they are employed clinically.

In conclusion, although the role of ion channel blockers in mycobacterial infections has been previously suggested, it remained unclear if these compounds have a direct effect on the bacteria, on the host cell, or on both entities. The new findings obtained in the current study validates our initial hypothesis that these compounds target both *M*. *tuberculosis* and the host macrophages and enable us to propose the following mechanism of action for these five compounds: a) in the bacteria, after entering, the compounds generate a cascade of events involving the inhibition of the respiratory chain complexes and energy production for efflux activity. Indirectly, this reduces the resistance level to antibiotics, potentiating their activity *in vitro*; b) on the host cell, the treatment with the ion channel blockers results in phagosome acidification and enhanced transcription of hydrolases, leading to bacterial growth restriction irrespective of their resistance pattern. Both effects cooperate and result in an enhanced killing activity that can be highly efficient when combined with antituberculosis drugs.

The combination of these compounds with tuberculosis chemotherapy can enhance antimycobacterial killing, prevent the emergence of resistance and reduce the duration of treatment thus presenting a novel strategy for tuberculosis therapy. Medicinal chemistry studies are now needed to improve the properties of these compounds, increasing their *M*. *tuberculosis* efflux-inhibition and killing-enhancement activity and reduce their toxicity for humans, therefore optimizing their potential for clinical usage [52]. This work further highlights the potential value of ion channel blockers, such as those evaluated in this study, as adjuvants of tuberculosis chemotherapy, in particular for the development of new therapeutic strategies, with strong potential for treatment shortening, an urgent demand in order to improve treatment and reduce drug resistance in tuberculosis.

## Supporting Information

S1 Fig Effect of the ion channel blockers against human monocyte-derived macrophages. Cells were treated with different concentrations of the compounds for 3 days; 10% of AlamarBlue was then added and the cells were further incubated during four hours at 37°C, 5% CO_2_. For the subsequent intracellular assays, the concentrations used were those that gave more than 90% macrophage viability (above the dashed line on each graph). A), verapamil; B), thioridazine; C), chlorpromazine; D), flupenthixol; and E), haloperidol.
(TIF)

S1 Table Primers used in the RT-qPCR assays. (DOCX)

S2 Table Genetic characterization of the efflux transporters studied. (DOCX)

S3 Table Concentrations required to inhibit *M. tuberculosis* in vitro versus that needed to produce similar effects on macrophages. (DOCX)
