# Supplementary figures and images for: Ion Channel Blockers as Antimicrobial Agents, Efflux Inhibitors, and Enhancers of Macrophage Killing Activity against Drug Resistant Mycobacterium tuberculosis
Source: PLoS One. 2016 Feb 26;11(2):e0149326. doi: 10.1371/journal.pone.0149326 (PMC4769142; doi:10.1371/journal.pone.0149326)

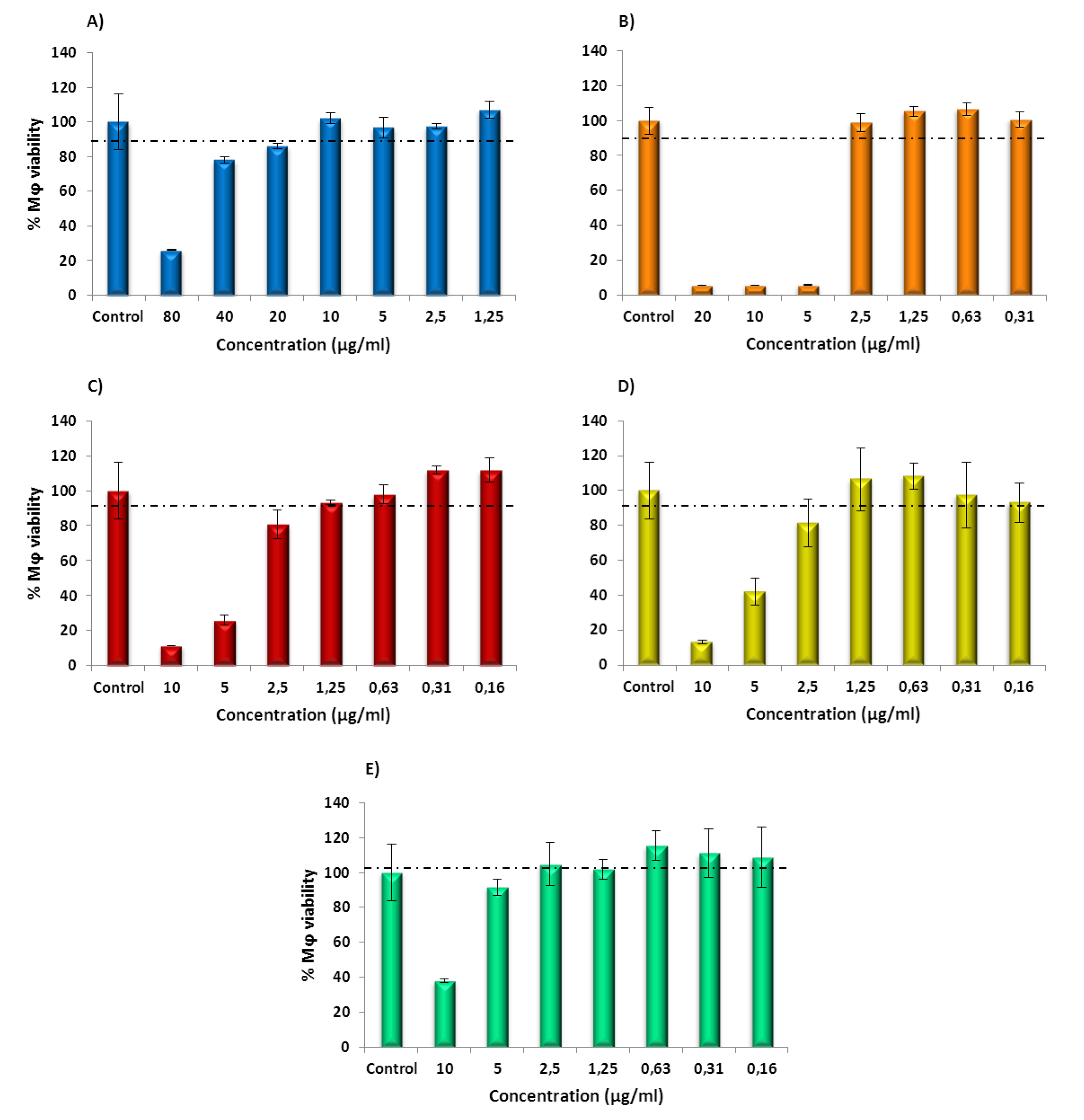

Supplement: S1 Fig — Cells were treated with different concentrations of the compounds for 3 days; 10% of AlamarBlue was then added and the cells were further incubated during four hours at 37°C, 5% CO2. For the subsequent intracellular assays, the concentrations used were those that gave more than 90% macrophage viability (above the dashed line on each graph). A), verapamil; B), thioridazine; C), chlorpromazine; D), flupenthixol; and E), haloperidol. (TIF) [file pone.0149326.s001.tif]
