# Supplementary material for: Ion Channel Blockers as Antimicrobial Agents, Efflux Inhibitors, and Enhancers of Macrophage Killing Activity against Drug Resistant Mycobacterium tuberculosis
Source: PLoS One. 2016 Feb 26;11(2):e0149326. doi: 10.1371/journal.pone.0149326 (PMC4769142; doi:10.1371/journal.pone.0149326)
Supplement: S1 Table — (DOCX) [file pone.0149326.s002.docx]

S1 Table. Primers used in the RT-qPCR assays.

| **Gene** | **Primer Sequence (5’-3’)** | **Amplification product (bp)** | **Reference** |
| --- | --- | --- | --- |
| *whiB7*_Fw | TCG AGG TAG CCA AGA CAC T | 109 | [This work] |
| *whiB7*_Rv | TCG AAT ATC TCA CCA CCC CA |  |  |
| *Rv2459*_Fw | CAT CTT CAT GGT GTT CGT G | 232 | [1] |
| *Rv2459*_Rv | CGG TAG CAC ACA GAC AAT AG |  |  |
| *mmpL7_*Fw | TAC CCA AGC TGG AAA CAA | 214 | [2] |
| *mmpL7*_Rv | CCG TCA GAA TAG AGG AAC CAG |  |  |
| *p55_*Fw | AGT GGG AAA TAA GCC AGT AA | 198 | [2] |
| *p55_*Rv | TGG TTG ATG TCG AGC TGT |  |  |
| *efpA_*Fw | ATG GTA ATG CCT GAC ATC C | 131 | [2] |
| *efpA_*Rv | CTA CGG GAA ACC AAC AAA G |  |  |
| *mmr_*Fw | AAC CAG CCT GCT CAA AAG | 221 | [2] |
| *mmr_*Rv | CAA CCA CCT TCA TCA CAG A |  |  |
| *Rv1258c*_Fw | AGT TAT AGA TCG GCT GGA TG | 268 | [2] |
| *Rv1258c*_Rv | GTG CTG TTC CCG AAA TAC |  |  |
| 16S*_*Fw | CAA GGC TAA AAC TCA AAG GA | 197 | [2] |
| 16S*_*Rv | GGA CTT AAC CCA ACA TCT CA |  |  |

**References**

1. Machado D, Couto I, Perdigão J, Rodrigues L, Portugal I, Baptista P, et al. Contribution of efflux to the emergence of isoniazid and multidrug resistance in *Mycobacterium tuberculosis*. PLoS One. 2012; 7 (4): e34538.
2. Rodrigues L, Machado D, Couto I, et al. Contribution of efflux activity to isoniazid resistance in the *Mycobacterium tuberculosis* complex. Infect Genet Evol. 2012; 12: 695-700.
