# Supplementary material for: Ion Channel Blockers as Antimicrobial Agents, Efflux Inhibitors, and Enhancers of Macrophage Killing Activity against Drug Resistant Mycobacterium tuberculosis
Source: PLoS One. 2016 Feb 26;11(2):e0149326. doi: 10.1371/journal.pone.0149326 (PMC4769142; doi:10.1371/journal.pone.0149326)
Supplement: S2 Table — (DOCX) [file pone.0149326.s003.docx]

S2 Table. Genetic characterization of the efflux transporters studied.

|  | **Strain** |  |  |  |  |  |  |
| --- | --- | --- | --- | --- | --- | --- | --- |
| **Gene** | **82/09** | **149/09** | **286/09** | **69/11** | **29/12** | **269/03** | **294/09** |
| ***iniA*** | del GCG after nt 285 | wt | del GCG after nt 285 | wt | wt | wt | wt |
| ***Rv1258c*** | wt | wt | wt | C194del | wt | wt | wt |
| ***p55*** | wt | Q506 | Q506 | Q506 | Q506 | wt | Q506 |
| ***Rv1217c*** | wt | A531 | A531 | A173T/S204/A531 | A531 | wt | wt |
| ***Rv1218c*** | wt | wt | wt | Q243R | wt | wt | wt |
| ***pstB*** | T61M | T61M | T61M | wt | T61M | wt | T61M |

Wt, wild type; del, deletion.
