# Supplementary material for: Ion Channel Blockers as Antimicrobial Agents, Efflux Inhibitors, and Enhancers of Macrophage Killing Activity against Drug Resistant Mycobacterium tuberculosis
Source: PLoS One. 2016 Feb 26;11(2):e0149326. doi: 10.1371/journal.pone.0149326 (PMC4769142; doi:10.1371/journal.pone.0149326)
Supplement: S3 Table — (DOCX) [file pone.0149326.s004.docx]

S3 Table. Concentrations required to inhibit *M. tuberculosis* *in vitro* *versus* that needed to produce similar effects on macrophages.

|  | **Concentration (µM)** | |
| --- | --- | --- |
| **Compound** | ***in vitro*** | ***ex vivo*** |
| **Verapamil** | 260 | 20.4 |
| **Thioridazine** | 18.4 | 6.1 |
| **Chlorpromazine** | 42.2 | 3.5 |
| **Flupenthixol** | 29.6 | 2.5 |
| **Haloperidol** | 79.8 | 3.3 |
